# Multiphase Kinetic Modeling of Air Pollutant Effects on Protein Modification and Nitrotyrosine Formation in Epithelial Lining Fluid

**DOI:** 10.1021/acs.est.3c03556

**Published:** 2023-08-17

**Authors:** Ashmi Mishra, Steven Lelieveld, Ulrich Pöschl, Thomas Berkemeier

**Affiliations:** Multiphase Chemistry Department, Max Planck Institute for Chemistry, Hahn-Meitner-Weg 1, 55128, Mainz, Germany

**Keywords:** Tyrosine, Epithelial lining fluid, Oxidants, Air pollution, Antioxidants, Oxidative stress

## Abstract

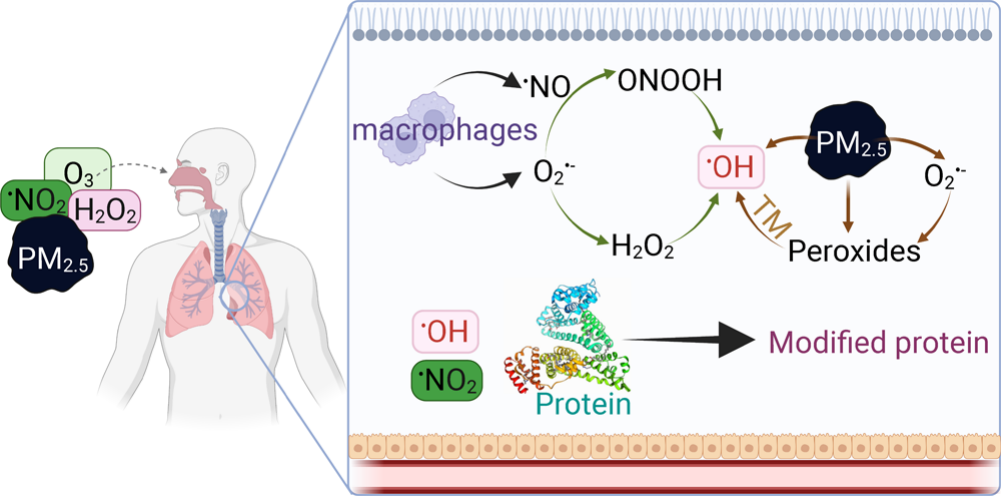

Exposure to ambient air pollution is a major risk factor for human health. Inhalation of air pollutants can enhance the formation of reactive species in the epithelial lining fluid (ELF) of the respiratory tract and can lead to oxidative stress and oxidative damage. Here, we investigate the chemical modification of proteins by reactive species from air pollution and endogenous biological sources using an extended version of the multiphase chemical kinetic model KM-SUB-ELF 2.0 with a detailed mechanism of protein modification. Fine particulate matter (PM_2.5_) and nitrogen dioxide (^•^NO_2_) act synergistically and increase the formation of nitrotyrosine (Ntyr), a common biomarker of oxidative stress. Ozone (O_3_) is found to be a burden on the antioxidant defense system but without substantial influence on the Ntyr concentration. In simulations with low levels of air pollution, the Ntyr concentration in the ELF is consistent with the range of literature values for bronchoalveolar lavage fluid from healthy individuals. With high levels of air pollution, however, we obtain strongly elevated Ntyr concentrations. Our model analysis shows how chemical reactions of air pollutants can modify proteins and thus their functionality in the human body, elucidating a molecular pathway that may explain air pollutant effects on human health.

## Abstract 2

Protein modification and nitrotyrosine formation in epithelial lining fluid are mediated by fine particulate matter and nitrogen dioxide.

## Introduction

Biological systems are subject to oxidants, including reactive oxygen species (ROS) and reactive nitrogen species (RNS). Oxidant production is essential to maintain cellular redox homeostasis,^1^ but an imbalance of oxidant production and antioxidant (AO) defense can lead to oxidative stress (distress).^2,3^ Oxidative damage to biological molecules is associated with development of diseases and aging processes.^4^ Both biological endogenous sources, such as mitochondria and phagocytic cells,^5,6^ as well as exogenous sources, such as air pollution and tobacco smoke, contribute to production of oxidants.^7−9^ Studies have shown elevated levels of oxidative damage in humans as a result of exposure to air pollution, which may cause increased oxidant production, decreased AO defense activity, or a combination of both.^10−12^

Exposure to ambient air pollution can cause adverse health outcomes and mortality.^13−15^ Fine particulate matter with a diameter less than 2.5 μm (PM_2.5_) and gaseous oxidants such as ozone (O_3_) and nitrogen dioxide (^•^NO_2_) are the most noxious components of air pollution, contributing to millions of excess deaths^16−18^ and emergency room visits annually.^19^

PM_2.5_ is made up of a variety of substances, including mineral dust, soot, and organic matter, and can come from a variety of natural and anthropogenic sources, such as residential energy use, power generation, break and tire wear, wildfires, and wind-blown dust.^20−23^ PM_2.5_ encompasses organic and inorganic compounds, including redox-active components such as transition metals, secondary organic aerosol (SOA), and quinones.^9,24,25^ Upon inhalation, PM_2.5_ generates reactive species in the respiratory tract, either directly through chemical reactions of redox-active components in PM_2.5_^9,26,27^ or by triggering oxidant production by cells such as neutrophils or macrophages.^28,29^

O_3_ exposure has been shown to contribute to risk of respiratory and circulatory mortality. The toxicity of O_3_ is displayed upon its inhalation and absorption into the respiratory tract.^30−32^ Through its strong oxidizing ability, O_3_ can react with biomolecular targets in the respiratory tract, such as proteins, lipids, and AOs.^33,34^ O_3_ can react with proteins, which make up a large fraction of biomolecules present in the respiratory tract, leading to protein modification.^35,36^

^•^NO_2_ originates mostly from combustion sources and atmospheric photochemistry.^23,37−39^ Exposure to high levels of ^•^NO_2_ can irritate the airways and cause respiratory problems, particularly in people with asthma and other pre-existing lung conditions.^40,41^ Long-term exposure to ^•^NO_2_ may be associated with an increased risk of mortality from respiratory and cardiovascular diseases;^42,43^ however, it is unclear if this is due to effects of ^•^NO_2_ itself or a confounder from co-emission alongside other harmful pollutants like PM_2.5_.^44−46^

Peroxynitrite (ONOO^–^) is a short-lived species that is formed in the fast reaction of ^•^NO and the superoxide radical (O_2_^•–^).^47−49^^•^NO is produced enzymatically in the body by nitric oxide synthase (NOS).^50^ O_2_^•–^ is ubiquitous in biological systems but is also formed in the respiratory tract upon inhalation of PM_2.5_.^9,26,29^ ONOO^–^ undergoes rapid reaction with carbon dioxide in biological systems, forming carbonate radicals (CO_3_^•–^) and ^•^NO_2_, both of which are one-electron oxidants.^51^ Alternatively, the protonated form of ONOO^–^, peroxynitrous acid (ONOOH), can decompose to the hydroxyl radical (^•^OH) and ^•^NO_2_.^47^

A mechanism by which air pollutants may cause adverse health outcomes is through the formation of oxidants, such as ROS and RNS, upon deposition in the epithelial lining fluid (ELF) of the respiratory tract.^9,52^ Highly reactive oxidants, such as ^•^OH and CO_3_^•–^, react rapidly with all biological molecules, such as DNA, cholesterol, lipids, carbohydrates, proteins, and AOs.^12,49,53^^•^OH has been identified as a major inducer of oxidative stress.^25^ In contrast, less-reactive oxidants, like hydrogen peroxide (H_2_O_2_), cause modifications at specific sites of biomolecules.^12,53^

Proteins are a major target of oxidants because of their high reactivity and their high abundance in cells and extracellular fluids such as the ELF.^12,25^ Gaseous and particulate air pollutants can interact with proteins and promote oxidation and nitration.^54,55^ For example, tyrosine residues can be oxidized by reactive species from air pollution (e.g., O_3_ and ^•^NO_2_)^36,56^ and endogenous biological sources (e.g., ONOO^–^),^49^ leading to the formation of tyrosyl radicals that can undergo nitration by ^•^NO_2_ and irreversibly form 3-nitrotyrosine (Ntyr).^48,57,58^ Increased concentrations of Ntyr have been detected in the bronchoalveolar lavage fluid of mice after inhalation exposure to PM_2.5_ but not after exposure to O_3_ alone.^59^ The post-translational modification of tyrosine can alter protein structure and function^48,49^ and has been proposed as a molecular rationale for the enhancement of allergic diseases by traffic-related air pollution.^58,60^

However, the interactions of multiple gas-phase and particulate pollutants with proteins in the respiratory tract have not been studied in detail previously. While there have been many studies that determined the reaction rates of oxidants with proteins,^12,35,53^ there is a lack of studies quantifying the influence of atmospheric air pollution on protein modification in the human respiratory tract. Further, the relative importance of exogenous atmospheric oxidants compared to endogenous biological oxidants on protein modification is unclear.

In this study, we extend the kinetic multilayer model of surface and bulk chemistry in the epithelial lining fluid of the respiratory tract (KM-SUB-ELF 2.0)^9,25,27^ by an explicit chemical mechanism of protein oxidation, including tyrosine modification. We aim to translate experimental studies into a mechanistic understanding of how air pollutants lead to protein oxidation. We quantify the chemical modification of proteins by oxidants from biological and atmospheric sources and compare our model results to measurements of nitrotyrosine as a marker for oxidative stress.

## Materials and Methods

### Kinetic Modeling

**Figure 1:**
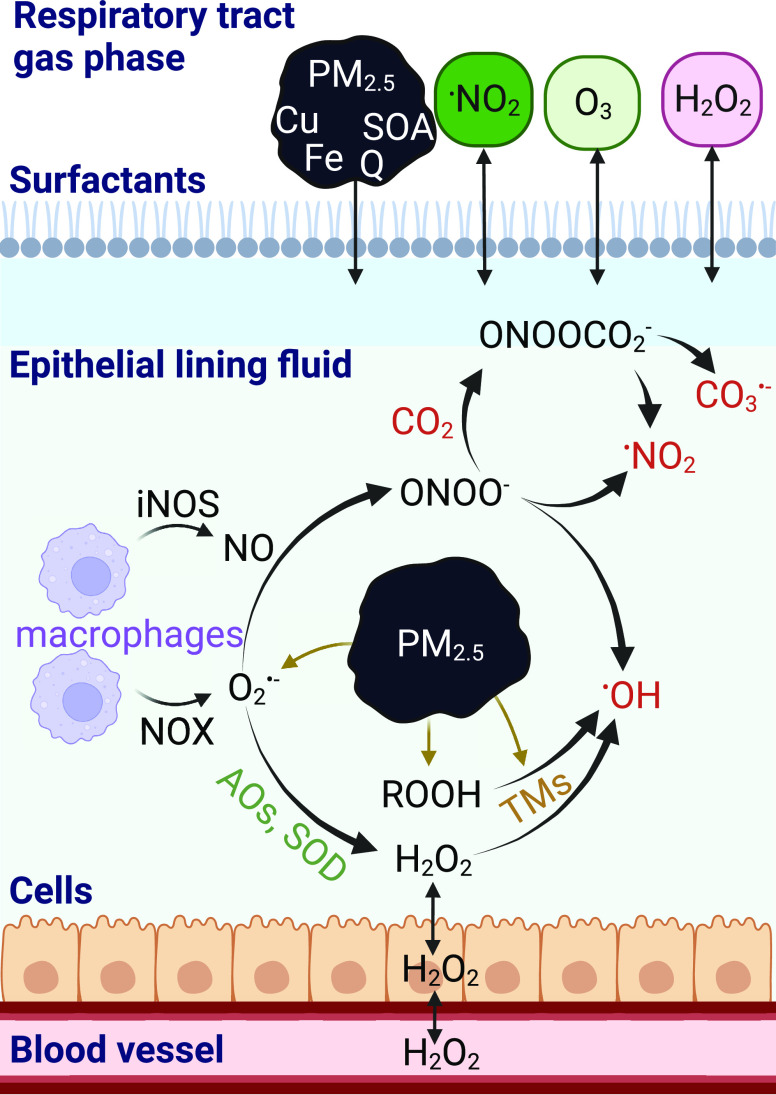
Pathways of endogenous and exogenous oxidant production that are included in the KM-SUB-ELF 2.0 model. The gaseous oxidants included in the model are ^•^NO_2_, H_2_O_2_, and O_3_. Endogenous sources produce superoxide (O_2_^•–^) which, in the presence of antioxidants (AOs) and enzymes, such as superoxide dismutase (SOD), can lead to the formation of hydrogen peroxide (H_2_O_2_). Transition metals (TMs) in PM_2.5_ catalyze the formation of hydroxyl radicals (^•^OH) through Fenton(-like) reactions of iron (Fe) with H_2_O_2_ or organic peroxides (ROOHs) contained within secondary organic aerosol (SOA). ^•^OH radicals are also formed by the decomposition of peroxynitrite (ONOO^–^), which is formed by the reaction of O_2_^•–^ and nitric oxide (^•^NO). ONOO^–^ reacts with CO_2_, decomposing to carbonate radicals CO_3_^•–^. Created with BioRender.com.

The kinetic model used in this study extends on the KM-SUB-ELF 2.0 model (Section S1).^9,25,27^ The model compartments include the respiratory tract gas phase, the surfactant layer, the aqueous ELF, a cellular layer, and a blood layer (Figures 1 and S1). The following processes in the model are explicitly resolved: inhalation, adsorption, and desorption of gas-phase molecules to and from the surfactant layer, diffusion between the surfactant layer, ELF, cells, and blood vessels, as well as a number of chemical reactions across the respiratory tract gas phase, surfactant layer, aqueous ELF, and the cellular layer.

The temporal evolution of reactants and reaction products is determined using a system of ordinary differential equations. In this study, the following air pollutants are considered: PM_2.5_ as a particulate pollutant (Section S2) as well as ^•^NO_2_, O_3_, and H_2_O_2_ as gaseous pollutants. The model simulates 2-h exposure scenarios during which the particulates are deposited in the ELF with a deposition fraction of 45%.^9,25,61,62^ For simplicity, the model does not account for the concentration gradient of gases or particles between the upper and lower sections of the respiratory system. Hence, the concentrations calculated in the model are averages over the entire respiratory tract.

A previously reported, standardized composition of PM_2.5_ is used in the model calculations. The composition was derived from median mass fractions of redox-active PM_2.5_ constituents in a large set of atmospheric field measurements.^25^ The mass fractions applied to all inhaled PM_2.5_ masses in the model are 3.1 × 10^–4^, 8.1 × 10^–3^, and 0.33 for copper, iron, and secondary organic aerosol (SOA), respectively, as well as 1.6 × 10^–5^ across three quinones: phenanthrenequinone, 1,2-naphthoquinone, and 1,4-naphthoquinone. The solubilities of the PM_2.5_ constituents copper and iron in the ELF were assumed to be 40% and 10%, respectively. Quinones and SOA are assumed to fully dissolve. Since PM_2.5_ and ^•^NO_2_ are often co-emitted, the gas-phase concentration of ^•^NO_2_ is co-varied with PM_2.5_ concentration with a factor of 1 μg m^–3 •^NO_2_ for each μg m^–3^ PM_2.5_ in the model simulations. O_3_ is treated with a fixed concentration of 30 ppb.

The following low molecular mass antioxidants (AOs) are included in the model: ascorbate (AscH), glutathione (GSH), uric acid (UAH), and α-tocopherol (α-Toc). Studies using healthy volunteers suggest that the AOs in the ELF do not fully deplete, despite exposure to 1 ppm ^•^NO_2_ for several hours.^63^ Therefore, the AO concentrations are fixed during the 2-h exposure simulation in this study to account for fast replenishment. The AscH, GSH, and UAH concentrations are 40 μM, 108 μM, and 200 μM, respectively.^64^ α-Toc is assumed to be in the surfactant layer only, with a concentration of 200 μM, which corresponds to a total ELF concentration of 0.7 μM.^64^ Other species included in the surfactant layer are surfactant lipid, 1-palmitoyl-2-oleoyl-*sn*-glycerol (POG), and surfactant protein (SP-B_1–25_). The reaction of O_3_ with POG in the surfactant layer produces H_2_O_2_ with a yield of 17% in the presence of water.^65,66^ Reactions of superoxide dismutase (SOD) and catalase are included in the ELF as enzymatic reactions,^67^ while in cells, a range of H_2_O_2_ scavenging enzymes (peroxiredoxins, catalase, GSH peroxidase) is considered (Section S3).

The model KM-SUB-ELF 2.0^9,25,27^ is extended in this study by inclusion of explicit reactions of amino acids in the ELF with various oxidants. Table S1 shows the full chemical mechanism used in this study. The model autogenerates a script based on an input chemical mechanism and consists of a system of differential equations. An autogenerated Jacobian matrix is used to accelerate and increase numerical stability of the differential equation solver (ODE23tb in MATLAB).

### Calculation of the Surface-Accessible Amino Acid Concentration

Some amino acids are buried within the protein and are not exposed on the surface, while others are on the surface and are, therefore, readily accessible for chemical reaction with dissolved molecules. Relative accessible surface area (RSA) is a measure of how exposed a particular amino acid is on the surface of a protein. We used Surface Racer^68^ to calculate RSA for amino acids present in human albumin as model protein. Human albumin is the protein with the highest mass fraction in the ELF.^69^ We calculate RSA by dividing the actual accessible surface area (ASA) of the amino acid by the maximum ASA that the amino acids could have if it were completely exposed on the surface of the protein (maxASA, eq 1). MaxASA values are available from previous studies that used Gly-X-Gly tripeptides, where X represents the amino acid residue of interest, as models.^70^ An RSA of 0 indicates that the amino acid is completely buried within the protein structure, and an RSA of 1 indicates that it is fully exposed on the surface.
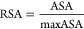
1

The mass of proteins within the ELF is approximately 10 mg/mL lung fluid. Based on the average molecular weight of an individual amino acid, which is about 125 g mol^–1^, the total concentration of amino acids in the ELF (*c*_AA_) is estimated to be around 80 mmol L^–1^. We use the RSA of every amino acid (RSA_*i*_), the total protein concentration *c*_AA_ divided by the total number of amino acids in the protein (*N*_AA_), and the number of the individual amino acids in the protein (*N*_AA,*i*_), to calculate the concentration of individual amino acids in the ELF (*c*_AA,*i*_).
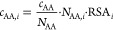
2

Hence, the model assumes that the reactivity of proteins is a linear combination of the reactivities of surface-accessible amino acids. The calculated surface-accessible amino acid concentrations are used as input parameters for the kinetic model and listed in Table S2.

## Results and Discussion

### Oxidants in Epithelial Lining Fluid

Inhalation of air pollution influences the endogenous baseline concentrations of oxidants in the epithelial lining fluid (ELF). The pathways of endogenous and exogenous oxidant production included in the kinetic model are presented in Figure 1. Superoxide (O_2_^•–^) is a key intermediate in many of these pathways. It is produced in the human body by NADPH oxidase (NOX) enzymes, which are highly expressed on the cell membrane of macrophages in the ELF.^6^ O_2_^•–^ is also formed through chemical reactions of redox-active components of air pollution (e.g., transition metals and quinones) in the ELF. Antioxidants (AOs) and enzymes, such as superoxide dismutase (SOD), transform O_2_^•–^ into hydrogen peroxide (H_2_O_2_). Due to its stability, H_2_O_2_ can diffuse through cell membranes and tissues. It is efficiently buffered by enzymes in the ELF but also converted into the highly reactive hydroxyl radical (^•^OH) through Fenton chemistry. The transition metals (TMs) necessary for Fenton reactions are inhaled through PM_2.5_. Secondary organic aerosol (SOA) contributes to the formation of ^•^OH in the ELF through Fenton-like reactions of organic peroxides (Section S4). Macrophages also produce nitric oxide (^•^NO) through the enzyme inducible nitric oxide synthase (iNOS). ^•^NO competes with SOD and AOs for the consumption of O_2_^•–^. The reaction of ^•^NO with O_2_^•–^ produces peroxynitrite (ONOO^–^), which in turn can react with CO_2_ to form carbonate radicals (CO_3_^•–^). The decomposition of ONOO^–^ is another pathway for the production of ^•^OH.

**Figure 2:**
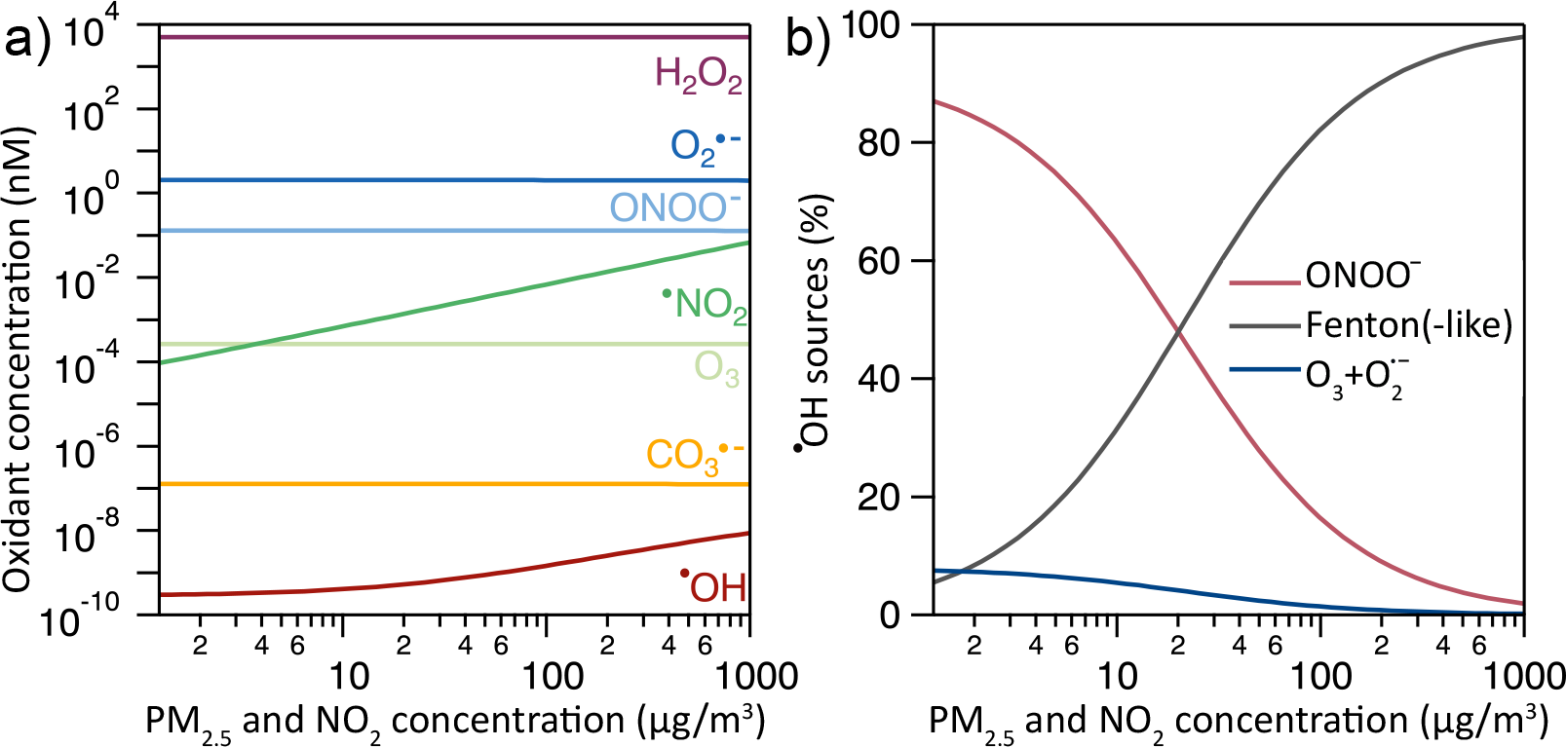
Oxidant concentration (a) and sources of ^•^OH (b) in the ELF as functions of ambient PM_2.5_ concentrations. Inhaled ^•^NO_2_ concentrations are varied alongside PM_2.5_ with a 1:1 mass ratio.

Figure 2 shows the concentration of oxidants in the ELF as a function of ambient PM_2.5_ and ^•^NO_2_ concentration. The model calculations show that H_2_O_2_ dominates the total concentration of oxidants. The other oxidants in the model, in descending order of their concentration, are O_2_^•–^, ONOO^–^, ^•^NO_2_, O_3_, CO_3_^•–^, and finally ^•^OH. The modeled H_2_O_2_ concentration in the ELF is higher than reported in previous studies,^9,25,27^ as a result of the inclusion of O_2_^•–^ production by alveolar macrophages (Section S5).^29^ The concentrations of ^•^NO_2_ and ^•^OH show an increasing trend with ambient PM_2.5_ and ^•^NO_2_, while the concentrations of O_3_, O_2_^•–^, H_2_O_2_, and CO_3_^•–^ remain constant. The ambient-gas-phase concentration of O_3_ is fixed in the model, which translates into the steady concentration of dissolved O_3_. For O_2_^•–^, H_2_O_2_, CO_3_^•–^, and ONOO^–^ the biological sources shown in Figure 1 dominate, as detailed in Figure S2. In the model, the biological production of O_2_^•–^ is parametrized according to the baseline production observed in Fang et al.^29^ using a rate of 2 × 10^14^ cm^–3^ s^–1^ (Section S5).

As shown in Figure 2b, ^•^OH production is mostly dependent on Fenton(-like) reactions involving PM_2.5_, the decomposition of ONOO^–^, and, to a lesser extent, the reaction of O_3_ and O_2_^•–^. At low PM_2.5_ and ^•^NO_2_ concentrations, the ONOO^–^ pathway of ^•^OH production dominates, while at high PM_2.5_ and ^•^NO_2_ concentrations, the Fenton chemistry dominates. At ∼25 μg m^–3^, the relative shares are roughly equal. Note that, when macrophages are exposed to PM_2.5_, higher rates of O_2_^•–^ production have been observed.^29^ Under such conditions, the ONOO^–^ pathway to ^•^OH production becomes more important in the model (Figure S3), which constitutes another PM-dependent ^•^OH production pathway.

### Reaction Pathways of Amino Acid Oxidation and Modification

Table 1 shows a compilation of reaction rate coefficients for the oxidation of amino acids with various oxidants. The ^•^OH radical reacts with all amino acid residues at a rate coefficient approaching the diffusion limit, while the less-reactive oxidants, such as H_2_O_2_ and O_2_^•–^, react only with certain amino acids. O_3_ reacts with all amino acids, albeit at a slower rate than ^•^OH. We note that, while CO_3_^•–^ likely reacts with more amino acids than indicated in Table 1, studies on this oxidant with other amino acids are lacking. However, since CO_3_^•–^ is of minor importance for the oxidation of tyrosine, cysteine, and methionine, the effect from CO_3_^•–^ on the oxidation of other amino acids may also be negligible.

**Table 1:** Second-Order Reaction Rate Coefficients (M^–1^ s^–1^) of Oxidants with Amino Acids

Amino acid	^•^OH	O_3_	CO_3_^•–^	ONOO^–^	H_2_O_2_	O_2_^•–^
Alanine	7.7 × 10^7,^^a^	1.0 × 10^3,^^c^				
Arginine	3.5 × 10^9,^^a^	5.3 × 10^3,^^c^				
Asparagine	4.9 × 10^7,^^a^	8.0 × 10^3,^^c^				
Aspartic acid	7.5 × 10^7,^^a^	6.2 × 10^2,^^c^				
Cysteine	3.4 × 10^10,^^a^	2.0 × 10^4,^^c^	2.0 × 10^8,^^e^	3.8 × 10^3^^f^	2.3^,^^f^	
Glutamine	5.4 × 10^8,^^a^	1.9 × 10^3,^^c^				
Glutamic acid	2.3 × 10^8,^^a^	8.8 × 10^2,^^c^				
Glycine	1.7 × 10^7,^^a^	3.5 × 10^3,^^c^				
Histidine	1.3 × 10^10,^^a^	1.7 × 10^4,^^c^				
Isoleucine	1.8 × 10^9,^^a^	9.8 × 10^2,^^c^				
Leucine	1.7 × 10^9,^^a^	9.6 × 10^2,^^c^				
Lysine	3.4 × 10^8,^^a^	3.5 × 10^3,^^c^				
Methionine	8.3 × 10^9,^^a^	3.8 × 10^6,^^c^	1.2 × 10^8,^^f^	3.6 × 10^2,^^f^	2.0 × 10^–2,^^f^	3.0 × 10^–1,^^f^
Phenylalanine	6.5 × 10^9,^^a^	1.9 × 10^4,^^c^				
Proline	4.8 × 10^8,^^a^	1.1 × 10^4,^^c^				
Serine	3.2 × 10^8,^^a^	8.0 × 10^3,^^c^				
Threonine	5.1 × 10^8,^^a^	3.6 × 10^3,^^c^				
Tryptophan	1.3 × 10^10,^^a^	7.0 × 10^6,^^c^		4.0 × 10^1,^^g^		
Tyrosine	1.2 × 10^10,^^b^	2.8 × 10^6,^^d^	4.5 × 10^7,^^g^			
Valine	7.6 × 10^8,^^a^	1.2 × 10^3,^^c^				

a Davies.^53^

b Solar et al.^71^

c Sharma and Graham.^35^

d Pryor et al.^72^

e Huie et al.^73^

f Davies.^12^

g Radi.^57^

Recent studies suggest that the catalytic production of ^•^OH radicals may be the main driver of the adverse health effects of PM_2.5_, rather than the chemical production of other oxidants such as H_2_O_2_.^27,74^ This explanation is plausible given the high reactivity of ^•^OH with the amino acids contained in proteins. Moreover, modified tyrosine residues in proteins, such as nitrotyrosine (Ntyr) and dityrosine (Dityr), are used as markers of oxidative stress.^10,11,75^ Thus, in this study, we focus on the oxidation chemistry of tyrosine.

**Figure 3:**
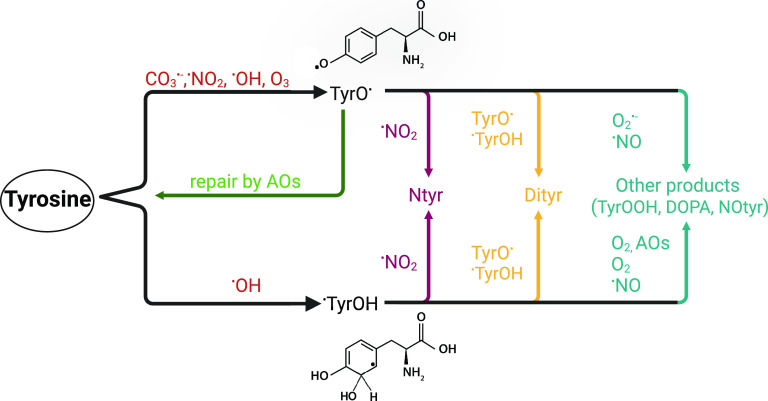
Pathways of tyrosine oxidation that are included in the KM-SUB-ELF 2.0 model. Oxidants (^•^OH, ^•^NO_2_, O_3_, and CO_3_^•–^) react with tyrosine, leading to the formation of tyrosyl radicals. TyrO^•^ radicals are repaired by antioxidants (AOs). Tyrosyl radicals react with ^•^NO_2_ to form nitrotyrosine (Ntyr). Two tyrosyl radicals react together to form dityrosine (Dityr). Reaction of tyrosyl radicals with O_2_ leads to the formation of other products, such as tyrosine peroxide (TyrOOH) and DOPA, while reaction with ^•^NO leads to the formation of nitrosotyrosine (NOtyr). Created with BioRender.com.

Figure 3 illustrates the main reaction pathways leading to modified tyrosine residues in the ELF. The reaction mechanism and rate coefficients of tyrosine modification in the model are presented in Table 2. The modification of tyrosine residues is a multistep process involving radical intermediates. There are several different biological, peroxynitrite-derived oxidants that oxidize tyrosine: ONOOH homolysis produces ^•^OH and ^•^NO_2_ radicals, while the reaction of ONOO^–^ with carbon dioxide leads to CO_3_^•–^. The ambient oxidants O_3_ and ^•^NO_2_, as well as ^•^OH formed from PM_2.5_, also oxidize tyrosine.^36,71,76^

**Table 2:** Second-Order Reaction Rate Coefficients (M^–1^ s^–1^) in the Chemical Mechanism of Tyrosine Modification Including Primary Reaction with Oxidants (^•^OH, O_3_, CO_3_^•–^, ^•^NO_2_), Secondary Reactions of Tyrosyl Radical Intermediates (TyrO^•^, ^•^TyrOH), and Repair by Antioxidants^a^

	Reaction	Rate coefficient (M^–1^ s^–1^)	Reference
1	Tyrosine + ^•^OH → ^•^TyrOH	1.2 × 10^10^	Solar et al.^71^
2	Tyrosine + ^•^OH → TyrO^•^	6.0 × 10^8^	Solar et al.^71^
3	Tyrosine + O_3_ → TyrO^•^	2.8 × 10^6^	Pryor et al.^72^
4	Tyrosine + CO_3_^•–^ → TyrO^•^	4.5 × 10^7^	Ferrer-Sueta et al.^49^
5	Tyrosine + ^•^NO_2_ → TyrO^•^	3.2 × 10^5^	Ferrer-Sueta et al.^49^
6	TyrO^•^ + ^•^NO_2_ → Ntyr	3.0 × 10^9^	Ferrer-Sueta et al.^49^
7	TyrO^•^ + ^•^NO → NOtyr	1.0 × 10^9^	Ferrer-Sueta et al.^49^
8	TyrO^•^ + TyrO^•^ → Dityr	2.3 × 10^8^	Ferrer-Sueta et al.^49^
9	TyrO^•^ + O_2_^•–^ → TyrOOH	1.5 × 10^9^	Ferrer-Sueta et al.^49^
10	^•^TyrOH + ^•^NO_2_ → Ntyr	3.0 × 10^9^	inferred from R6
11	^•^TyrOH + ^•^NO → NOtyr	1.0 × 10^9^	inferred from R7
12	^•^TyrOH + ^•^TyrOH → Dityr	3.0 × 10^8^	Solar et al.^71^
13	^•^TyrOH + TyrO^•^ → Dityr	2.3 × 10^8^	Ferrer-Sueta et al.^49^
14	^•^TyrOH + O2 → ^•^OOTyrOH	1.0 × 10^3^	Candeias et al.^77^
15	TyrO^•^ + GSH → Tyrosine	2.0 × 10^6^	Folkes et al.^78^
16	TyrO^•^ + AscH → Tyrosine	4.4 × 10^8^	Hunter et al.^79^
17	TyrO^•^ + UAH → Tyrosine	2.4 × 10^8^	Ferrer-Sueta et al.^49^
18	^•^OOTyrOH + AscH → TyrOOH	7.0 × 10^6^	Alfassi et al.^80^
19	^•^OOTyrOH + UAH → TyrOOH	1.9 × 10^6^	Alfassi et al.^80^

a Glutathione, GSH; ascorbic acid, AscH; uric acid, UAH.

Oxidation of tyrosine forms tyrosyl radicals in two ways: H abstraction on the hydroxyl group leads to a phenoxyl radical (TyrO^•^),^36,71,81^ while the addition of ^•^OH to the ring produces a ^•^TyrOH radical.^49,82,83^ During tyrosine nitration, both radicals react with ^•^NO_2_ in a radical—radical recombination reaction to Ntyr.^76,84,85^ Tyrosyl radicals also give rise to other modification products. Reaction of two tyrosyl radicals may form dityrosine (Dityr).^71,79,81^ Other products include TyrOOH, which is produced in the reaction of ^•^TyrOH with O_2_ and AOs^12,77,86,87^ or the reaction of TyrO^•^ with O_2_^•–^.^81,88,89^ The reaction of ^•^TyrOH with O_2_, followed by elimination of a hydroperoxyl group, can also lead to the formation of 3,4-dihydroxyphenylalanine (DOPA).^82^ Note that we currently do not differentiate between these two reaction pathways in the model as the branching ratio is unknown; therefore, DOPA is implicitly considered in TyrOOH in this study. Another product considered in the model is nitrosotyrosine (NOtyr), which is formed from the reaction of both tyrosyl radicals with ^•^NO (Figure S4).

**Figure 4:**
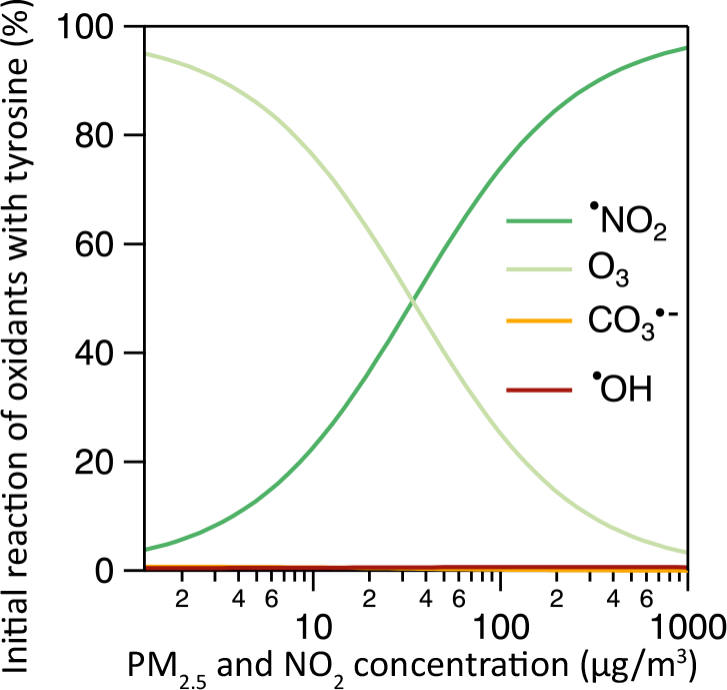
Contribution of oxidants to tyrosyl radical formation as a function of the ambient pollutant concentrations. Inhaled ^•^NO_2_ concentrations are varied alongside PM_2.5_ with a 1:1 mass ratio. The calculations assume a fixed O_3_ concentration of 30 ppb and a biological O_2_^•–^ production rate of 2 × 10^14^ cm^–3^ s^–1^.

Figure 4 shows the contribution of the oxidants (^•^NO_2_, O_3_, CO_3_^•–^ and ^•^OH) to the initial step of tyrosine modification (formation of tyrosyl radicals) as a function of PM_2.5_ and ^•^NO_2_. The model shows that at low ^•^NO_2_ concentrations, tyrosine reacts almost exclusively with O_3_, while reactions with ^•^NO_2_, ^•^OH, and CO_3_^•–^ are minor. At higher ambient concentrations, ^•^NO_2_ increasingly contributes to the oxidation of tyrosine and is the dominant oxidant at very high levels. At roughly 25 μg m^–3^ PM_2.5_ and ^•^NO_2_, tyrosine reacts with O_3_ and ^•^NO_2_ in equal quantities. Here, ^•^OH and CO_3_^•–^ radicals only contribute about 2%. This is due to the much higher concentrations of O_3_ and ^•^NO_2_ compared to those of ^•^OH and CO_3_^•–^, as shown in Figure 2a.

**Figure 5:**
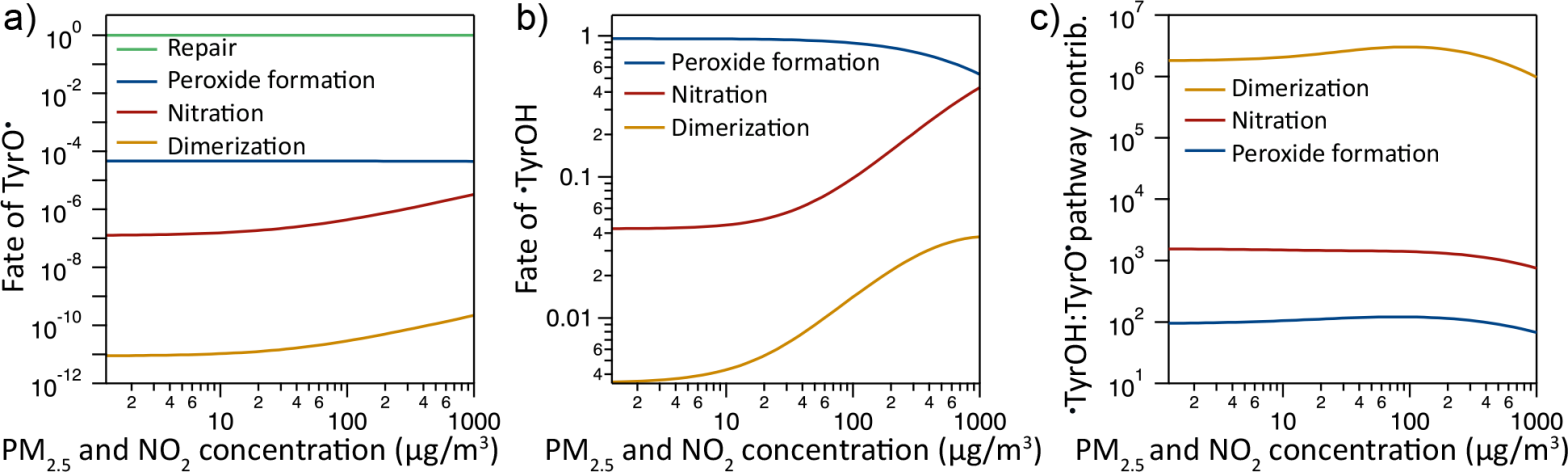
Reaction pathways of tyrosyl radicals in the chemical mechanism. Contribution of different reaction pathways to the fate of (a) TyrO^•^ and (b) ^•^TyrOH as a function of PM_2.5_ and ^•^NO_2_ in the ELF. (c) The ratio of modified tyrosine products generated through the ^•^TyrOH pathway to the amount generated through the TyrO^•^ pathway.

Figure 5 shows the competing reactions of the two different tyrosyl radicals as a function of the PM_2.5_ and ^•^NO_2_ concentrations. The model shows that almost all TyrO^•^ produced in the ELF are scavenged by antioxidants (AOs) under full repair (Figure 5a).^76,79^ At very high concentrations of PM_2.5_ and ^•^NO_2_, there is a slight increase in the formation of Ntyr and Dityr from TyrO^•^, but the repair pathway still dominates. For the ^•^TyrOH radical, however, a repair pathway has not been reported in the literature to our knowledge. An in vitro study even found an increase in the formation of DOPA when adding AscH.^90^ DOPA is associated with the ^•^OH-derived radical ^•^TyrOH.^82^ Therefore, in the model, the ^•^OH-induced formation of ^•^TyrOH always leads to modification of tyrosine.

The simulation shows that peroxide formation is the dominant reaction pathway over nitration and dimerization (Figure 5b). This is due to the much higher steady-state concentration of O_2_ in the ELF compared to that of ^•^NO_2_ and Tyr^•^. At low pollutant concentrations, the formation of Ntyr and Dityr does not show a dependence on PM_2.5_ and ^•^NO_2_ concentrations. At high pollutant concentrations, however, increasing PM_2.5_ and ^•^NO_2_ directly increases the fraction of ^•^TyrOH forming Ntyr and Dityr. This is because only above a PM_2.5_ and ^•^NO_2_ concentration of 25 μg m^–3^, the production of ^•^OH becomes dominated by PM_2.5_ (Figure 2b). The production of Ntyr is higher than that of Dityr due to the low steady-state concentration of the ^•^TyrOH radical.

Figure 5c shows that, despite the low steady-state concentration of ^•^OH, and thus a much lower fraction of tyrosine reacting with ^•^OH, modification of tyrosine occurs predominantly via ^•^TyrOH. Thus, although the initial attack to tyrosine by O_3_ and ^•^NO_2_ is much higher than by ^•^OH (shown in Figure 4), ^•^OH is largely responsible for the formation of modified tyrosine (Figure S5). ^•^OH is formed in reactions involving transition metals contained in particulate matter (Figure 2b). Hence, this result is consistent with the findings of Kelly and Fussell, who compiled studies linking air pollution exposure to markers of oxidative stress and found increased Ntyr concentrations after exposure to particulate matter.^91^ In contrast, the simulation results suggest that O_3_ is less involved in the formation of Ntyr due to the efficient repair of the tyrosyl radicals derived from O_3_.

### Influence of Individual Air Pollutants on Tyrosine Modification

**Figure 6:**
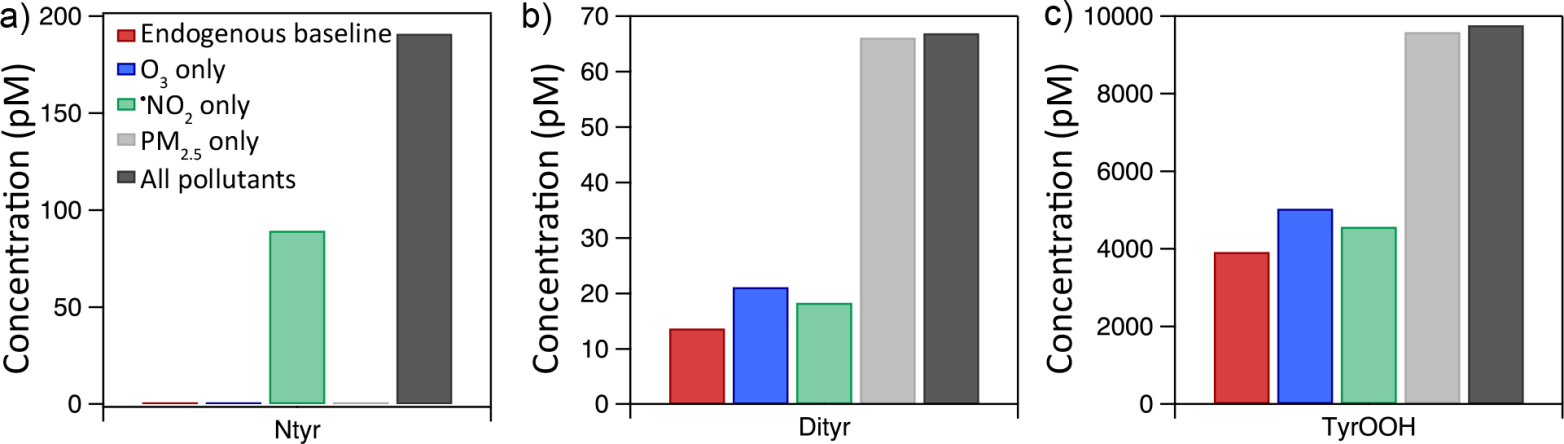
Concentration of modified tyrosine species in the ELF after the 2-h exposure in different pollution scenarios in KM-SUB-ELF 2.0. The contribution of individual pollutants (O_3_, PM_2.5_, and ^•^NO_2_) is evaluated by simulation of exposure to only the single pollutant, a scenario without pollutants (endogenous baseline, red), and a scenario with all pollutants (black). Panels a, b, and c show results for three tyrosine modifications, nitrotyrosine, dityrosine, and tyrosine peroxide, respectively. Note that in panel a, the *y*-axis is slightly offset for visualization purposes.

Figure 6 breaks down the individual contributions of air pollutants to the modification of tyrosine. The pollutant concentrations are derived from a standard pollution scenario (25 μg m^–3^ PM_2.5_, 25 μg m^–3 •^NO_2_, and 30 ppb O_3_). The biological sources of ^•^NO and O_2_^•–^ lead to an endogenous baseline concentration (red bar). Panel a shows the production of Ntyr from PM_2.5_ (gray), O_3_ (blue), and ^•^NO_2_ (green). Simulations were carried out using only the single pollutant. A scenario including all pollutants (dark gray) is shown for comparison. The single pollutant that is most correlated with the formation of Ntyr is ^•^NO_2_, followed by PM_2.5_ and then O_3_.

Note that endogenous ONOO^–^ plays a key role in both PM_2.5_- and ^•^NO_2_-only scenarios. In the PM_2.5_-only scenario, the ^•^NO_2_ required for nitration is solely produced from the decomposition of ONOO^–^ and thus becomes a limiting factor for the production of Ntyr. In the same regard, for the ^•^NO_2_-only scenario, ^•^OH is only produced from the decomposition of ONOO^–^ and, in this case, becomes a limiting factor for the initial ^•^TyrOH formation. The “all pollutant” scenario shows a higher production of Ntyr compared to the sum of only PM_2.5_ or only ^•^NO_2_ scenarios suggesting that PM_2.5_ and ^•^NO_2_ show synergistic effects toward the formation of Ntyr, i.e., Ntyr production depends nonlinearly on the presence of both PM_2.5_ and ^•^NO_2_. The synergistic effect contributing to the simulated Ntyr concentrations is quantified for a wide range of PM_2.5_ and ^•^NO_2_ concentrations in the Supporting Information (Figure S6).

Traffic-related air pollution encompasses a wide range of gases and particles resulting from the use of motor vehicles, particularly ^•^NO_2_ and PM_2.5_.^46,92,93^ Meta analyses show that long-term exposure to ^•^NO_2_ and PM_2.5_ has been associated with adverse health effects.^46,94^ Thus, the synergistic formation of Ntyr by exposure to ^•^NO_2_ and PM_2.5_ is consistent with the epidemiological evidence and may give indications why the co-emission of ^•^NO_2_ and PM_2.5_ from traffic-related sources leads to particularly negative health outcomes.

Panel b shows the same analysis for the formation of Dityr. The PM_2.5_-only scenario shows much higher Dityr production than the baseline scenario and is comparable to that of the scenario involving all pollutants. This is due to the additional production of ^•^OH by PM_2.5_, which increases the steady-state concentration of ^•^TyrOH. The O_3_-only scenario exhibits a slightly higher Dityr production compared with the baseline scenario. This is due to the reaction of O_3_ with O_2_^•–^, which contributes roughly 3.5% to the total ^•^OH production under these conditions (Figure 2b). ^•^NO_2_ does not lead to an increase in Dityr production.

Panel c shows the contribution of the individual air pollutants to the other products considered in the model. The total concentrations of other products (i.e., TyrOOH) are much higher than the concentrations of Ntyr and Dityr, reaching roughly 9 nM in the pollution scenario considered here. As seen for Dityr in panel b, the PM_2.5_-only scenario has a much higher contribution to tyrosine modification compared to the other individual pollutants.

Note that compared to Ntyr, the baseline values for Dityr and other tyrosine modification products are significantly higher. This is because the ^•^NO_2_ required for Ntyr formation is much more efficiently scavenged by AOs than ^•^OH radicals. ^•^OH radicals are continuously produced from the decomposition of ONOO^–^, leading to a baseline production of Dityr and other modification products. The low endogenous baseline and pronounced increase in concentration is a favorable property of Ntyr as a marker substance assessing oxidative stress from exposure to air pollution.

We also note that protein hydroperoxides such as TyrOOH may be labile and produce free radicals in secondary reactions. In reactions with enzymes (e.g., catalase), the hydroperoxides may react to the more stable alcohols, but in the presence of transition metals, Fenton-like chemistry may generate alkoxy radicals and ^•^OH.^87^ As the rate coefficients of these reactions are rather uncertain, a detailed chemical kinetic analysis of such products is beyond the scope of this article and will be addressed in future studies. Instead, we perform a sensitivity analysis for the reaction of protein peroxides with catalase (Figure S7) by assuming that TyrOOH reacts with the same rate coefficient as reported for hydrogen peroxide in the literature and found that model results are insensitive. Therefore, enzymes may not easily repair the peroxide. This is consistent with an experimental study in which about 40% of protein hydroperoxides decayed spontaneously in 24 h.^87,95^ Thus, in the 2-h exposure window considered in the model, the decay might be minor.

### Markers of Oxidative Stress in BAL Fluid

**Figure 7:**
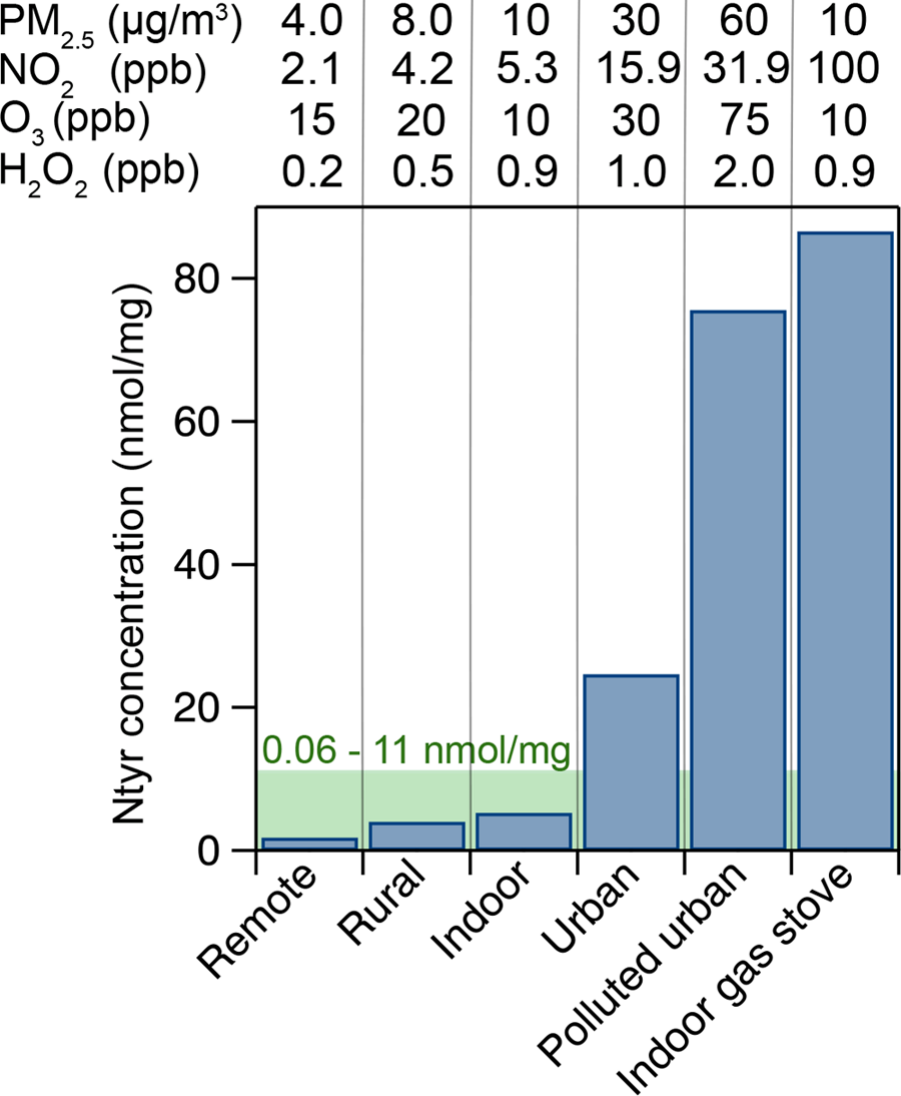
Concentration of nitrotyrosine (Ntyr) after the 2-h exposure to air pollution under different pollution scenarios in KM-SUB-ELF 2.0. The green shaded region shows Ntyr concentrations derived from the extraction of bronchoalveolar lavage (BAL) fluid.^10,11^ The pollutant concentrations are chosen based on measurements reported in the literature (Table S3).^66,101−114^

Figure 7 shows the Ntyr concentrations in different pollution scenarios (i.e., ambient concentrations of PM_2.5_, ^•^NO_2_, and O_3_) in the model (Table S3). We observe that less-polluted conditions typical for remote and rural areas, as well as indoor air, lead to low Ntyr concentrations of 1.8–5.2 nmol/mg. These numbers are consistent with experimental studies of Ntyr in bronchoalveolar lavage (BAL) fluid.^10,11^ BAL is a procedure used to determine the composition of the ELF. However, the ELF is diluted by the lavage procedure, and a dilution factor, often calculated from the concentration of urea, is used to infer concentrations in the ELF of the respiratory tract.^96^ When correcting for the dilution process during the extraction of BAL fluid, the measurement values correspond to concentrations of Ntyr of 0.6–11 nmol/mg in the ELF. In more-polluted conditions typical for urban locations, however, the modeled Ntyr concentrations are strongly elevated at 24.7–75.6 nmol/mg and thus exceed the concentrations typically found in the ELF.

Gas stoves and ovens emit significant amounts of ^•^NO_2_.^97−99^ In indoor environments where gas stoves are used, particularly in kitchens with inadequate ventilation, inhaled ^•^NO_2_ concentrations commonly reach 100 ppb.^98^ Using such a high concentration of ^•^NO_2_ in the indoor air scenario, the model yields a Ntyr concentration that is comparable to that of the polluted urban scenario.

We note that the turnover time of the ELF and therefore the half-life of modified proteins in the ELF are uncertain and influence the computed and experimentally derived values. A practical consequence of a rapid turnover of the ELF would be that test subjects in polluted areas might quickly adopt Ntyr concentrations that are typical for indoor concentrations of air pollution before a BAL procedure. Other sources of uncertainty in the model include endogenous production rates of ROS and concentrations and activity of antioxidant enzymes, as well as reaction rate coefficients involving peroxides from secondary organic aerosol (SOA).^25,27^ Furthermore, there is variability in the literature concerning the levels of antioxidants in the ELF. For instance, Rahman et al.^100^ report concentrations that differ from the values presented by van der Vliet et al.^64^ and used in KM-SUB-ELF. Antioxidants represent the largest sink of ^•^NO_2_ in the model. The uncertainty in Ntyr production from the choice of antioxidant concentrations is in the range of a factor of 2 (Figure S8). Moreover, the deposition fraction of PM_2.5_ depends on the shape of the particle size distribution. The deposition fraction of 45% used here is a value typical for 1 μm particles.^61,62^ A lower limit for the deposition fraction is 20%, which yields Ntyr concentrations that are ∼40% lower (Figure S9). Some uncertainty may arise from concentration gradients of particles and gases along the respiratory tract. Using an extension to KM-SUB-ELF that is currently under development, we estimate that losses of O_3_ and ^•^NO_2_ in the upper respiratory tract could reduce their concentrations in the lower respiratory tract by ∼15%. Together with a (re)distribution of deposited particles, this could translate into a reduction of model-predicted Ntyr concentrations by ∼35% (Figure S10). Overall, the Ntyr concentrations determined in this study should be seen as order-of-magnitude estimate. Future studies will be needed to reduce the model uncertainty.

By linking reaction kinetics with observed markers of oxidative stress, this study provides a mechanistic understanding of the chemistry by which air pollution may affect human health. We find that the permanent modification of tyrosine residues is mostly caused by initial oxidation with the ^•^OH radical, which is generated in processes involving particulate pollutants (PM_2.5_). The gas-phase pollutants O_3_ and ^•^NO_2_ also oxidize tyrosine, but the resulting intermediates are efficiently repaired by antioxidants according to the model simulations. Thus, this pathway may only be a burden on the antioxidant defense rather than causing irreversible chemical modification of proteins. According to the model, the air pollutants most responsible for the formation of nitrotyrosine are PM_2.5_ and ^•^NO_2_, which are also the two air pollutants most commonly associated with increased mortality from traffic-related air pollution. These insights into the specific interactions and differential toxicity of individual air pollutants provide a foundation and direction for further research on the adverse health effects of air pollution.

## Supporting Information Available

Overview KM-SUB-ELF 2.0 (S1); Particulate pollutant concentrations in the ELF (S2); Enzyme concentrations and reactions (S3); Parameterization of ROS formation from secondary organic aerosol (S4); Endogenous superoxide production from macrophages (S5); Chemical reactions (Table S1); Input parameters of the KM-SUB-ELF 2.0 model (Table S2); Concentrations of pollutants used in the pollution scenarios (Table S3); Schematic overview of KM-SUB-ELF 2.0 (Figure S1); Biological vs atmospheric contribution of various oxidants explored in the study (Figure S2); Contribution of different sources to ^•^OH production in the model as a function of endogenous superoxide production (Figure S3); Modified tyrosine concentration in the ELF as a function of the concentration of three distinct pollutants (Figure S4); Fraction of modified tyrosine attributed to different tyrosyl radicals (Figure S5); Nitrotyrosine concentration in the epithelial lining fluid as a function of PM_2.5_ and ^•^NO_2_ levels (Figure S6); Sensitivity of tyrosine peroxide concentration to decomposition reaction with catalase (Figure S7); Concentration of nitrotyrosine and dityrosine as a function of antioxidant concentrations (Figure S8); Sensitivity of PM_2.5_ deposition fractions to concentration of Ntyr after 2-h exposure to air pollution under different pollution scenarios (Figure S9); Concentration of nitrotyrosine in the gas phase of the upper and lower respiratory tract (Figure S10) (PDF)

## Abbreviations Used

Dityr: Dityrosine
Ntyr: Nitrotyrosine
TyrOOH: Tyrosine peroxide
PM_2.5_: Particulate matter
ROS: Reactive oxygen species
BAL: Bronchoalveolar lavage
