# Supplementary material for: Multiphase Kinetic Modeling of Air Pollutant Effects on Protein Modification and Nitrotyrosine Formation in Epithelial Lining Fluid
Source: Environ Sci Technol. 2023 Aug 17;57(34):12642–53. doi: 10.1021/acs.est.3c03556 (PMC10469477; doi:10.1021/acs.est.3c03556)
Supplement: Supplementary file 1 — es3c03556_si_001.pdf [file es3c03556_si_001.pdf]

Supplementary Materials for

Multiphase kinetic modelling of air pollutant effects on protein modification and nitrotyrosine formation in epithelial lining fluid

Ashmi Mishra[1], Steven Lelieveld[1], Ulrich Pöschl[1], Thomas Berkemeier[1,*]

Affiliations:

[1]Multiphase Chemistry Department, Max Planck Institute for Chemistry; Mainz, Germany.

*Correspondence to: Thomas Berkemeier (t.berkemeier@mpic.de)

Contents of Supporting Information

Additional information

S1: Overview KM-SUB-ELF 2.0

S2: Particulate pollutant concentrations in the ELF

S3: Enzyme concentrations and reactions

S4: Parameterization of ROS formation from secondary organic aerosol

S5: Endogenous superoxide production from macrophages

S6: Synergistic effect of $^{\bullet}NO_2$ and $PM_{2.5}$

Tables

Table S1: Chemical reactions

Table S2: Input parameters of the KM-SUB-ELF 2.0 model

Table S3: Concentrations of pollutants used in the pollution scenarios

Figures

Figure S1: Schematic overview of KM-SUB-ELF 2.0

Figure S2: Biological vs. atmospheric contribution of various oxidants explored in the study

Figure S3: Contribution of different sources to $^{\bullet}OH$ production in the model as a function of endogenous superoxide production

Figure S4: Modified tyrosine concentration in the ELF as a function of the concentration of three distinct pollutants

Figure S5: Fraction of modified tyrosine attributed to different tyrosyl radicals

Figure S6: Nitrotyrosine concentration in the epithelial lining fluid as a function of $PM_{2.5}$ and $^{\bullet}NO_2$ levels

Figure S7: Sensitivity of tyrosine peroxide concentration to decomposition reaction with catalase

Figure S8: Concentration of nitrotyrosine and dityrosine as a function of antioxidant concentrations

Figure S9: Sensitivity of $PM_{2.5}$ deposition fractions to concentration of Ntyr after 2 h exposure to air pollution under different pollution scenarios

Figure S10: Concentration of nitrotyrosine in the gas phase of the upper and lower respiratory tract

Supplementary Text

S1. Overview KM-SUB-ELF 2.0

The kinetic multi-layer model of surface and bulk chemistry in the epithelial lining fluid (KM-SUB-ELF 2.0)[1] describes chemical reactions and mass transport in the respiratory tract, linking atmospheric and physiological chemistry, with the goal of understanding chemical mechanism behind the adverse health effects or air pollution. Table S1 shows the chemical mechanism used in this study, which includes redox chemistry of transition metals, HO_x and NO_x radical chemistry, antioxidant redox reaction as well as oxidation reactions of proteins in a total of 201 reactions. Of these, 23 are gas-phase chemical reactions adopted from the Master Chemical Mechanism (MCM),[2,3] and 178 are aqueous-phase reactions within the ELF, of which six occur in the surfactant layer, and two in the cellular layer.

S2. Particulate pollutant concentrations in the ELF

The concentrations of redox-active $PM_{2.5}$ constituents in the ELF are calculated as described previously,[4] using Eq. S1.

$$C_{\mathrm{ELF,Y}} = \frac{C_{\mathrm{gas,PM2.5}} \times Q \times t_{\mathrm{acc}} \times f_{\mathrm{dep,PM2.5}} \times w_{\mathrm{Y}} \times s_{\mathrm{Y}}}{M_{\mathrm{Y}} \times V_{\mathrm{ELF}}} \quad \text{(Eq. S1)}$$

The initial concentrations of redox-active $PM_{2.5}$ constituents in the ELF ($C_{ELF,Y}$) are calculated based on ventilation rate (Q), deposition fraction ($f_{dep,PM2.5}$), accumulation time in the ELF (t_{acc}), fractional solubilities (s_Y), and their mass fractions in $PM_{2.5}$ (w_Y). The mass fractions are median values from field observations across 70 different sites across the globe. The median mass fractions of Fe, Cu, SOA and the three quinone species are 8.14×10^{-3}, 3.07×10^{-4}, 0.333, 6.28×10^{-6}, 3.43×10^{-6} and, 6.64×10^{-6}, respectively. As fractional solubilities, s_Y, we use 0.4 and 0.1 for copper and iron ions, respectively. For the lung ventilation rate, we assume 1.5 $m^3\ h^{-1}$, for $f_{dep,PM2.5}$ we assume 0.45 of all the inhaled $PM_{2.5}$, and the model simulates a 2-hour exposure episode (t_{acc}). The total ELF volume, V_{ELF}, is set to 20 mL.

S3. Enzyme concentrations and reactions

The enzyme activity in the ELF is applied as previously described.[4] Briefly, the molar concentrations of enzymes are calculated from enzyme activity in enzyme units (*U*), the catalytic constant, k_{cat}, and Eq. S2.

$$[\text{Enzyme}] = \frac{v_{max}}{k_{cat}} \qquad \text{(Eq. S2)}$$

One *U* is defined as the amount of enzyme needed to catalyze 1 micromole of substrate per minute. Catalase (CAT) is an important endogenous H_2O_2 scavenger in the ELF.[9] $U_{cat,ELF}$ is 3.7 ± 0.6 *U* mL^{-1},[9] and $k_{cat,CAT}$ is reported to range between 10^5 – 10^6 s^{-1}. These values translate into a molar concentration ranging from 1.3 – 24 pM (~5 pM) in the ELF. The glutathione peroxidase (GPx) concentration in the model ELF is 50 nM based on a molar mass of 2.19×10^4 g mol^{-1} and a mass concentration of 1 µg mL^{-1} in ELF.[10] In cells, the catalytic activity of catalase is reported to be an order of magnitude higher compared to ELF, which equates to 50 pM.[11,12] Peroxiredoxins are thiol proteins that are present in the cells at much higher concentrations than catalase, with concentrations of tens of micromolar.[13] Hence, peroxiredoxins are probably the predominant sink of H_2O_2 in lung cells. For simplicity, we choose a conservative estimate of 10 µM for the sum of all H_2O_2-scavenging enzymes in this work, and assume a common reaction rate of 3.3×10^{-14} cm^3 s^{-1} for the enzymes.[13] Two superoxide ($^{\bullet}O_2^-$) molecules are converted into H_2O_2 and O_2 by superoxide dismutase (SOD; Tab. S1, Reaction No. 127). SOD exists in cells in micromolar levels hence, using the same scaling factor as for H_2O_2 between cells and ELF, we have an ELF SOD concentration of 0.1 uM. We note that *U* and k_{cat} are difficult to determine because the ELF is very difficult to sample. We therefore note that the enzyme concentrations used in this study are subject to uncertainty.

S4. Parameterization of ROS formation from secondary organic aerosol

Secondary organic aerosol (SOA) has been shown to form reactive oxygen species (ROS) in aqueous solutions and the process is included in the model.[5–7] Because the exact reaction mechanism is unclear, ROS formation by SOA in the ELF is parameterized using formation rates

of $^{\bullet}OH$ based on experimental observations.[5,6,8] Tong et al. (2016) quantified $^{\bullet}OH$ production of SOA, from which we infer first- and second-order rate coefficients that reproduce a molar yield in the experimental data of 0.1% in the absence of iron (R137; Tab. S1), and a 1% yield of $^{\bullet}OH$ in the presence of iron (R138; Tab. S1), respectively.

S5. Endogenous superoxide production from macrophages

A recent study shows that macrophages contribute to formation of ROS in the ELF, by releasing superoxide.[14] Fang et al. show a baseline production superoxide rate of about 0.06 µM min-1 in experiments using 4×10^4 cells cm^{-3}.[14] A macrophage concentration in the ELF of 1.5×10^7 cm^{-3} has been reported [9], which is used to linearly scale the observed superoxide production rate.

$$P_{O_2^{\bullet -}} = 0.06\ \mu\mathrm{M\ min}^{-1} \cdot \frac{1.5 \cdot 10^7\ \mathrm{cm}^{-3}}{4 \cdot 10^4\ \mathrm{cm}^{-3}} = 22.5\ \mu\mathrm{M\ min}^{-1} = 2.26 \cdot 10^{14}\ \mathrm{cm}^{-3}\ \mathrm{s}^{-1}$$

We use a rounded value of $2.0 \cdot 10^{14}$ cm^{-3} s^{-1} as endogenous superoxide production rate from macrophages in the aqueous epithelial lining fluid layer in the model.

S6. Synergistic effect of $^{\bullet}NO_2$ and $PM_{2.5}$

To illustrate the synergistic effect of $^{\bullet}NO_2$ and $PM_{2.5}$, we calculate the concentration of nitrotyrosine as a function of $^{\bullet}NO_2$ and $PM_{2.5}$ (Fig. S8a) and determine the percentage increase due to synergy (i.e. exceeding the expected linear additive behavior) according to Eq. S3.

$$\text{synergy} = \frac{[\mathrm{Ntyr}]_{\mathrm{PM}_{2.5}\&\mathrm{NO}_2} - [\mathrm{Ntyr}]_{\mathrm{PM}_{2.5}\ \mathrm{only}} - [\mathrm{Ntyr}]_{\mathrm{NO}_2\ \mathrm{only}}}{[\mathrm{Ntyr}]_{\mathrm{PM}_{2.5}\ \mathrm{only}} + [\mathrm{Ntyr}]_{\mathrm{NO}_2\ \mathrm{only}}} \cdot 100 \qquad \text{(Eq. S3)}$$

Here, $[\mathrm{Ntyr}]_{\mathrm{PM}_{2.5}\&\mathrm{NO}_2}$ means the concentration of nitrotyrosine in a calculation containing both $PM_{2.5}$ and $^{\bullet}NO_2$. $[\mathrm{Ntyr}]_{\mathrm{PM}_{2.5}\ \mathrm{only}}$ is the nitrotyrosine concentration at $^{\bullet}NO_2$ = 0 ppb, and vice versa.

The results are shown in Fig. S8b. We find a substantial synergistic effect that is mostly a function of $PM_{2.5}$ concentration. At a $PM_{2.5}$ concentration > 10 µg/m^3, the synergistic effect always exceeds 20 %. At very high pollutant concentrations, however, it can reach up to 3000 %, i.e. the synergistic effect is 30x bigger than the linear effect.

Supplementary Tables

Table S1. Chemical reactions used in the KM-SUB-ELF 2.0 model, with reference.

#	Reaction	Rate constant (cm^{-3} s^{-1} or s^{-1})	Ref.
	Gas-phase reactions		
**1**	$^{\bullet}NO + O_3 \rightarrow {}^{\bullet}NO_2 + O_2$	2.05×10^{-14}	2,3
**2**	$^{\bullet}NO_2 + O_3 \rightarrow NO_3^{\bullet} + O_2$	4.85×10^{-17}	2,3
**3**	$^{\bullet}NO + {}^{\bullet}NO + O_2 \rightarrow {}^{\bullet}NO_2 + {}^{\bullet}NO_2$	8.93×10^{-20}	2,3
**4**	$^{\bullet}NO + NO_3^{\bullet} \rightarrow {}^{\bullet}NO_2 + {}^{\bullet}NO_2$	2.57×10^{-11}	2,3
**5**	$^{\bullet}NO_2 + NO_3^{\bullet} \rightarrow {}^{\bullet}NO + {}^{\bullet}NO_2 + O_2$	7.73×10^{-16}	2,3
**6**	$^{\bullet}NO_2 + NO_3^{\bullet} \rightarrow N_2O_5$	1.21×10^{-12}	2,3
**7**	$^{\bullet}OH + O_3 \rightarrow HO_2^{\bullet} + O_2$	8.20×10^{-14}	2,3
**8**	$^{\bullet}OH + H_2O_2 \rightarrow HO_2^{\bullet} + H_2O$	1.73×10^{-12}	2,3
**9**	$HO_2^{\bullet} + O_3 \rightarrow {}^{\bullet}OH + O_2 + O_2$	8.24×10^{-16}	2,3
**10**	$^{\bullet}OH + HO_2^{\bullet} \rightarrow H_2O + O_2$	1.08×10^{-10}	2,3
**11**	$HO_2^{\bullet} + HO_2^{\bullet} \rightarrow H_2O_2 + O_2$	5.09×10^{-12}	2,3
**12**	$HO_2^{\bullet} + O_2^{\bullet -} + H^{+} \rightarrow H_2O_2 + O_2$	3.50×10^{-12}	2,3
**13**	$^{\bullet}OH + {}^{\bullet}NO \rightarrow HONO$	8.91×10^{-12}	2,3
**14**	$^{\bullet}OH + NO_2^{\bullet} \rightarrow HNO_3$	8.91×10^{-12}	2,3
**15**	$^{\bullet}OH + NO_3^{\bullet} \rightarrow HO_2^{\bullet} + {}^{\bullet}NO_2$	2.00×10^{-11}	2,3
**16**	$HO_2^{\bullet} + {}^{\bullet}NO \rightarrow {}^{\bullet}OH + {}^{\bullet}NO_2$	8.24×10^{-12}	2,3
**17**	$HO_2^{\bullet} + {}^{\bullet}NO_2 \rightarrow HO_2NO_2$	6.87×10^{-13}	2,3
**18**	$HO_2NO_2 \rightarrow HO_2^{\bullet} + {}^{\bullet}NO_2$	2.49×10^{-1}	2,3
**19**	$^{\bullet}OH + HO_2NO_2 \rightarrow {}^{\bullet}NO_2 + H_2O + O_2$	2.96×10^{-12}	2,3
**20**	$HO_2^{\bullet} + {}^{\bullet}NO_3^{\bullet} \rightarrow {}^{\bullet}OH + {}^{\bullet}NO_2 + O_2$	4.00×10^{-12}	2,3
**21**	$^{\bullet}OH + HONO \rightarrow {}^{\bullet}NO_2 + H_2O$	5.78×10^{-12}	2,3
**22**	$^{\bullet}OH + HNO_3 \rightarrow NO_3^{\bullet} + H_2O$	1.37×10^{-13}	2,3
**23**	$N_2O_5 \rightarrow {}^{\bullet}NO_2 + NO_3^{\bullet}$	1.83×10^{-1}	2,3
	Surfactant reactions		
**24**	SPB + $^{\bullet}OH \rightarrow$ SPB-ox	1.70×10^{-11}	15–17
**25**	POG + $^{\bullet}OH \rightarrow$ POG-ox	1.70×10^{-11}	8
**26**	SPB + $O_3 \rightarrow$ SPB-ox	1.00×10^{-14}	18,19
**27**	POG + $O_3 \rightarrow$ POG-ox + 0.17 H_2O_2	1.66×10^{-16}	20–23
**28**	aToc + OH $\rightarrow$ aToc-ox	4.50×10^{-13}	24
**29**	aToc + $O_3 \rightarrow$ aToc-ox	1.20×10^{-18}	25
	Aqueous ELF reactions		
**30**	$O_2^{\bullet -} + HO_2 + H_2O \rightarrow H_2O_2 + OH^{-} + O_2$	1.70×10^{-13}	8,26
**31**	$HO_2 + HO_2 \rightarrow H_2O_2 + O_2$	1.40×10^{-15}	26
**32**	$O_2^{\bullet -} + O_2^{\bullet -} + 2H^{+} \rightarrow H_2O_2 + O_2$	3.82×10^{-16}	26
**33**	$H_2O_2 + {}^{\bullet}OH \rightarrow HO_2 + H_2O$	5.50×10^{-14}	27

**34**	$^{\bullet}OH + {}^{\bullet}OH \rightarrow H_2O_2$	8.60×10^{-12}	28
**35**	$^{\bullet}OH + O_2^{\bullet-} \rightarrow O_2 + OH^-$	1.30×10^{-11}	15
**36**	$^{\bullet}OH + HO_2 \rightarrow H_2O + O_2$	1.20×10^{-11}	28
**37**	$H_2O_2 + HO_2 \rightarrow {}^{\bullet}OH + O_2 + H_2O$	4.98×10^{-21}	29
**38**	$Fe^{2+} + O_2^{\bullet-} + 2H^+ \rightarrow Fe^{3+} + H_2O_2$	3.10×10^{-14}	8,26
**39**	$Fe^{2+} + HO_2 + H^+ \rightarrow Fe^{3+} + H_2O_2$	1.99×10^{-15}	30
**40**	$Fe^{2+} + H_2O_2 \rightarrow Fe^{3+} + {}^{\bullet}OH + OH^-$	4.30×10^{-18}	31
**41**	$Fe^{2+} + {}^{\bullet}OH \rightarrow Fe^{3+} + OH^-$	5.30×10^{-13}	32
**42**	$Fe^{2+} + H_2O_2 \rightarrow Fe^{4+} + H_2O$	9.50×10^{-18}	8
**43**	$Fe^{3+} + H_2O_2 \rightarrow Fe^{2+} + HO_2 + H^+$	3.32×10^{-24}	31
**44**	$Fe^{3+} + HO_2 \rightarrow Fe^{2+} + O_2 + H^+$	3.30×10^{-18}	26
**45**	$Fe^{3+} + O_2^{\bullet-} \rightarrow Fe^{2+} + O_2 + H^+$	3.30×10^{-18}	26
**46**	$Fe^{4+} + Fe^{2+} \rightarrow Fe^{3+} + Fe^{3+}$	6.60×10^{-18}	33
**47**	$Fe^{3+} + AscH \rightarrow Fe^{2+} + Asc^{\bullet}$	1.10×10^{-19}	8
**48**	$Fe^{4+} + AscH \rightarrow Fe^{3+} + Asc^{\bullet}$	7.60×10^{-19}	8
**49**	$Fe^{2+} + O_2 \rightarrow O_2^{\bullet-} + Fe^{3+}$	5.20×10^{-21}	8
**50**	$Cu^+ + HO_2 + H^+ \rightarrow Cu^{2+} + H_2O_2$	2.30×10^{-12}	8
**51**	$Cu^+ + O_2^{\bullet-} + H_2O \rightarrow Cu^{2+} + H_2O_2 + OH^-$	5.80×10^{-15}	8
**52**	$Cu^{2+} + HO_2 \rightarrow Cu^+ + O_2 + H^+$	1.60×10^{-11}	8
**53**	$Cu^{2+} + O_2^{\bullet-} \rightarrow Cu^+ + O_2$	8.30×10^{-12}	8
**54**	$Cu^{2+} + AscH \rightarrow Cu^+ + Asc^{\bullet}$	1.40×10^{-18}	8
**55**	$Cu^+ + O_2 \rightarrow Cu^{2+} + O_2^{\bullet-}$	6.90×10^{-20}	8
**56**	$Cu^+ + H_2O_2 \rightarrow Cu^{2+} + {}^{\bullet}OH + OH^-$	2.40×10^{-20}	8
**57**	$Cu^+ + H_2O_2 \rightarrow Cu^{3+} + OH^- + OH^-$	5.00×10^{-19}	8
**58**	$Cu^+ + Cu^{3+} \rightarrow Cu^{2+} + Cu^{2+}$	5.80×10^{-12}	8
**59**	$Cu^{2+} + H_2O_2 \rightarrow Cu^+ + O_2^{\bullet-} + H^+$	3.80×10^{-24}	8
**60**	$PQN + AscH \rightarrow PQN^{\bullet} + Asc^{\bullet}$	1.20×10^{-20}	34
**61**	$PQN^{\bullet} + O_2 \rightarrow PQN + O_2^{\bullet-}$	4.60×10^{-13}	8
**62**	$PQN^{\bullet} + O_2^{\bullet-} + 2H^+ \rightarrow PQN + H_2O_2$	3.30×10^{-12}	8
**63**	$NQN12 + AscH \rightarrow NQN12^{\bullet} + Asc^{\bullet}$	1.50×10^{-19}	34
**64**	$NQN12^{\bullet} + O_2 \rightarrow NQN12 + O_2^{\bullet-}$	4.60×10^{-13}	8
**65**	$NQN12^{\bullet} + O_2^{\bullet-} + 2H^+ \rightarrow NQN12 + H_2O_2$	3.30×10^{-12}	8
**66**	$NQN14 + AscH \rightarrow NQN14^{\bullet} + Asc^{\bullet}$	6.30×10^{-21}	34
**67**	$NQN14^{\bullet} + O_2 \rightarrow NQN14 + O_2^{\bullet-}$	4.60×10^{-13}	8
**68**	$NQN14^{\bullet} + O_2^{\bullet-} + 2H^+ \rightarrow NQN14 + H_2O_2$	3.30×10^{-12}	8
**69**	$UAH + O_3 \rightarrow Products$	2.35×10^{-15}	35
**70**	$UAH + {}^{\bullet}OH \rightarrow Products + OH^-$	1.20×10^{-11}	36
**71**	$GSH + {}^{\bullet}OH \rightarrow Products + OH^-$	1.50×10^{-11}	37
**72**	$GSSG + {}^{\bullet}OH \rightarrow Products + OH^-$	1.50×10^{-11}	4
**73**	$Asc^{\bullet} + Asc^{\bullet} + H^+ \rightarrow AscH + DHA$	5.00×10^{-16}	38
**74**	$AscH + O_2^{\bullet-} + H^+ \rightarrow Asc^{\bullet} + H_2O_2$	5.10×10^{-17}	8

**75**	$AscH + HO_2 \rightarrow Asc^{\bullet} + H_2O_2$	2.65×10^{-17}	39
**76**	$AscH + {}^{\bullet}OH \rightarrow Products + OH^-$	1.80×10^{-11}	40
**77**	$AscH + O_3 \rightarrow Products$	9.97×10^{-14}	35
**78**	$GS^- + O_3 \rightarrow Products$	1.33×10^{-12}	35
**79**	$GSH + O_3 \rightarrow Products$	1.33×10^{-12}	35
**80**	$GSOO^{\bullet} + GSOO^{\bullet} \rightarrow 0.56\ O_2^{\bullet -} + Products$	6.79×10^{-13}	41
**81**	$O_2^{\bullet -} + GSH \rightarrow GSO^{\bullet} + OH^-$	3.32×10^{-19}	42–44
**82**	${}^{\bullet}NO_2 + GS^{\bullet} \rightarrow GSNO_2$	4.98×10^{-12}	45
**83**	$GSOO^{\bullet} + {}^{\bullet}NO_2 \rightarrow GSOONO_2$	2.49×10^{-12}	41
**84**	$GSOONO_2 \rightarrow GSOO^{\bullet} + {}^{\bullet}NO_2$	7.5×10^{-1}	41
**85**	${}^{\bullet}NO_2 + GS^- \rightarrow NO_2^- + GS^{\bullet}$	4.00×10^{-13}	45
**86**	${}^{\bullet}NO_2 + GSH \rightarrow NO_2^- + GS^{\bullet} + H^+$	1.66×10^{-14}	46
**87**	$GSOO^{\bullet} + GSH \rightarrow GSO^{\bullet} + GSOH$	3.32×10^{-15}	45
**88**	$GSO + NO_2 \rightarrow GSOONO$	7.47×10^{-12}	45
**89**	$GSOONO \rightarrow Products$	7.00×10^{2}	45
**90**	$GS^{\bullet} + GS^- \rightarrow GSSG^{\bullet -}$	1.59×10^{-14}	45,47
**91**	$GS^{\bullet} + O_2 \rightarrow GSOO^{\bullet}$	3.20×10^{-12}	47
**92**	$GSOO^{\bullet} \rightarrow GS^{\bullet} + O_2$	6.00×10^{5}	47
**93**	$GSSG^{\bullet -} \rightarrow GS^{\bullet} + GS^-$	1.60×10^{5}	45,47
**94**	$GSSG^{\bullet -} + O_2 \rightarrow GSSG + O_2^{\bullet -}$	8.30×10^{-12}	45,47
**95**	$GS^{\bullet} + GS^{\bullet} \rightarrow GSSG$	8.30×10^{-12}	47
**96**	$GSOH + GSH \rightarrow GSSG + H_2O$	1.20×10^{-18}	48
**97**	$GSO^{\bullet} + GSO^{\bullet} \rightarrow Products$	9.96×10^{-14}	45
**98**	$GS^- + H_2O_2 \rightarrow GSOH + OH^-$	1.60×10^{-21}	48
**99**	$GS^{\bullet} + AscH \rightarrow GSH + Asc^{\bullet}$	1.00×10^{-12}	49,50
**100**	$UA + {}^{\bullet}NO_2 \rightarrow UA^{\bullet} + NO_2^-$	3.00×10^{-14}	51,52
**101**	$AscH + {}^{\bullet}NO_2 \rightarrow Asc^{\bullet} + NO_2^-$	5.80×10^{-14}	51,52
**102**	$UA^{\bullet} + AscH \rightarrow UA + Asc^{\bullet}$	1.70×10^{-15}	50
**103**	$GS^{\bullet} + UA \rightarrow GSH + UA^{\bullet}$	5.00×10^{-14}	46
**104**	$O_2^{\bullet -} + {}^{\bullet}NO_2 \rightarrow O_2NOOm$	7.50×10^{-12}	45,53
**105**	$O_2NOO^- \rightarrow NO_2^- + O_2$	7.00×10^{-1}	45
**106**	$O_2NOO^- \rightarrow O_2^{\bullet -} + {}^{\bullet}NO_2$	1.10×10^{0}	45
**107**	${}^{\bullet}NO_2 + {}^{\bullet}NO_2 \rightarrow N_2O_4$	7.50×10^{-13}	54
**108**	$N_2O_4 \rightarrow {}^{\bullet}NO_2 + {}^{\bullet}NO_2$	6.90×10^{3}	54
**109**	$N_2O_4 + H_2O \rightarrow NO_2^- + NO_3^- + 2H^+$	1.00×10^{3}	45
**110**	$O_2^{\bullet -} + O_3 + H_2O \rightarrow {}^{\bullet}OH + 2O_2 + OH^-$	2.50×10^{-12}	55
**111**	$HO_2 + O_3 \rightarrow {}^{\bullet}OH + 2O_2$	1.66×10^{-17}	55
**112**	$NO_2^- + {}^{\bullet}OH \rightarrow {}^{\bullet}NO_2 + OH^-$	8.80×10^{-12}	53
**113**	${}^{\bullet}OH + {}^{\bullet}NO_2 \rightarrow NO_3^- + H^+$	7.50×10^{-12}	45
**114**	${}^{\bullet}OH + {}^{\bullet}NO_2 \rightarrow ONOOH$	7.50×10^{-12}	45
**115**	$ONOOH \rightarrow {}^{\bullet}NO_2 + {}^{\bullet}OH$	3.00×10^{-1}	45

**116**	$ONOOH \rightarrow NO_3^- + H^+$	7.00×10^{-1}	[45]
**117**	$ONOO^- + GSH \rightarrow NO_2^- + GSOH$	1.10×10^{-18}	[56]
**118**	$GSO^\bullet + {}^\bullet NO_2 \rightarrow GSOONO$	7.50×10^{-12}	[45]
**119**	$GSOONO + H_2O \rightarrow$ Products	7.00×10^{2}	[45]
**120**	$ONOOH + AscH \rightarrow Im_1$	1.66×10^{-15}	[57]
**121**	$Im_1 \rightarrow ONOOH + AscH$	5.00×10^{2}	[57]
**122**	$Im_1 \rightarrow Im_2$	4.00×10^{1}	[57]
**123**	$Im_2 \rightarrow Im_1$	5.00×10^{0}	[57]
**124**	$Im_2 + AscH \rightarrow Asc + DHA + NO_2^- + H_2O$	1.66×10^{-19}	[57]
**125**	$Im_2 \rightarrow Asc + NO_3^- + H^+$	8.50×10^{-1}	[57]
**126**	$ONOOH + UA \rightarrow UA^{rad} + NO_2$ + Products	2.60×10^{-19}	[58]
**127**	$O_2^{\bullet -} + SOD \xrightarrow{2H^+} H_2O_2 + SOD$	2.65×10^{-12}	[4]
**128**	H_2O_2 + catalase $\rightarrow H_2O + 0.5\ O_2$ + catalase	3.20×10^{-14}	[4]
**129**	$ONOOH$ + Glutathione peroxidase $\rightarrow NO_2^-$	1.33×10^{-14}	[59]
**131**	$O_2^{\bullet -} + {}^\bullet NO \rightarrow ONOO^-$	3.16×10^{-11}	[60]
**132**	$ONOO^- + CO_2 \rightarrow ONOOCO_2^-$	4.98×10^{-17}	[61]
**133**	$ONOOCO_2^- \rightarrow {}^\bullet NO_2 + CO_3^{\bullet -}$	3.3×10^{5}	[45,61]
**134**	$ONOOCO_2^- \rightarrow NO_3^- + CO_2$	6.7×10^{5}	[45,61]
**135**	$ONOO^- + GSH \rightarrow NO_2^- + GSOH$	2.24×10^{-18}	[62]
**136**	${}^\bullet NO_2 + {}^\bullet NO \rightarrow N_2O_3$	1.83×10^{-12}	[61]
**137**	$SOA \rightarrow {}^\bullet OH$	5.56×10^{-7}	*See SI text*
**138**	$SOA + Fe^{2+} \rightarrow {}^\bullet OH + Fe^{3+}$	7.90×10^{-23}	*See SI text*
**139**	$Ala + O_3 \rightarrow$ products	1.66×10^{-18}	[63]
**140**	$Arg + O_3 \rightarrow$ products	8.80×10^{-18}	[63]
**141**	$Asp + O_3 \rightarrow$ products	1.03×10^{-18}	[63]
**142**	$Cys + O_3 \rightarrow$ products	3.32×10^{-17}	[63]
**143**	$Glu + O_3 \rightarrow$ products	1.46×10^{-18}	[63]
**144**	$Gly + O_3 \rightarrow$ products	5.81×10^{-18}	[63]
**145**	$His + O_3 \rightarrow$ products	2.82×10^{-17}	[63]
**146**	$Ile + O_3 \rightarrow$ products	1.63×10^{-18}	[63]
**147**	$Leu + O_3 \rightarrow$ products	1.59×10^{-18}	[63]
**148**	$Lys + O_3 \rightarrow$ products	5.81×10^{-18}	[63]
**149**	$Met + O_3 \rightarrow$ products	6.31×10^{-15}	[63]
**150**	$Phe + O_3 \rightarrow$ products	3.16×10^{-17}	[63]
**151**	$Pro + O_3 \rightarrow$ products	1.83×10^{-17}	[63]
**152**	$Ser + O_3 \rightarrow$ products	1.33×10^{-17}	[63]
**153**	$Thr + O_3 \rightarrow$ products	5.98×10^{-18}	[63]
**154**	$Trp + O_3 \rightarrow$ products	1.16×10^{-14}	[63]
**155**	$Val + O_3 \rightarrow$ products	1.99×10^{-18}	[63]
**156**	$Ala + {}^\bullet OH \rightarrow$ products	1.28×10^{-13}	[64]
**157**	$Arg + {}^\bullet OH \rightarrow$ products	5.81×10^{-12}	[64]

**158**	Asp + $^{\bullet}OH$ → products	1.25×10^{-13}	64
**159**	Cys + $^{\bullet}OH$ → products	5.65×10^{-11}	64
**160**	Glu + $^{\bullet}OH$ → products	3.82×10^{-13}	64
**161**	Gly + $^{\bullet}OH$ → products	2.82×10^{-14}	64
**162**	His + $^{\bullet}OH$ → products	2.16×10^{-11}	64
**163**	Ile + $^{\bullet}OH$ → products	2.99×10^{-12}	64
**164**	Leu + $^{\bullet}OH$→ products	2.82×10^{-12}	64
**165**	Lys + $^{\bullet}OH$ → products	5.65×10^{-13}	64
**166**	Met + $^{\bullet}OH$ → products	1.38×10^{-11}	64
**167**	Phe + $^{\bullet}OH$ → products	1.08×10^{-11}	64
**168**	Pro + $^{\bullet}OH$ → products	7.97×10^{-13}	64
**169**	Ser + $^{\bullet}OH$ → products	5.31×10^{-13}	64
**170**	Thr +$^{\bullet}OH$ → products	8.47×10^{-13}	64
**171**	Trp + $^{\bullet}OH$ → products	2.16×10^{-11}	64
**172**	Val + $^{\bullet}OH$ → products	1.26×10^{-12}	64
**173**	Cys + $CO_3^{\bullet-}$ → products	1.99×10^{-13}	65
**174**	Met + $CO_3^{\bullet-}$ → products	1.99×10^{-13}	66
**175**	Cys + $ONOO^-$ → products	6.31×10^{-18}	66
**176**	Met + $ONOO^-$ → products	5.98×10^{-19}	66
**177**	Trp + $ONOO^-$ → products	6.64×10^{-20}	120
**178**	Cys + H_2O_2 → products	3.82×10^{-21}	66
**179**	Met + H_2O_2 → products	3.32×10^{-23}	66
**180**	Met + $O_2^{\bullet-}$ → products	4.98×10^{-22}	66
**181**	Tyrosine + O3 → TyrO$^{\bullet}$	4.65×10^{-15}	19
**182**	Tyrosine + $^{\bullet}OH$ → TyrO$^{\bullet}$	9.96×10^{-13}	67
**183**	Tyrosine + $^{\bullet}NO_2$→ TyrO$^{\bullet}$	7.47×10^{-14}	68
**184**	Tyrosine + $CO_3^{\bullet-}$ → TyrO$^{\bullet}$	5.31×10^{-16}	68
**185**	Tyrosine + $^{\bullet}OH$ → $^{\bullet}$TyrOH	2.00×10^{-11}	67
**186**	TyrO$^{\bullet}$ + $^{\bullet}NO_2$ → Ntyr	4.98×10^{-12}	68
**187**	$^{\bullet}$TyrOH + $^{\bullet}NO_2$→ Ntyr	4.98×10^{-12}	*Same as 186*
**188**	TyrO$^{\bullet}$ + $^{\bullet}NO$ → NOtyr	1.66×10^{-12}	68
**189**	$^{\bullet}$TyrOH + $^{\bullet}NO$ → NOtyr	1.66×10^{-12}	*Same as 188*

**190**	$TyrO^{\bullet} + TyrO^{\bullet} \rightarrow Dityr$	3.82×10^{-13}	67
**191**	$^{\bullet}TyrOH + {}^{\bullet}TyrOH \rightarrow Dityr$	4.98×10^{-13}	67
**192**	$^{\bullet}TyrOH + TyrO^{\bullet} \rightarrow Dityr$	3.82×10^{-13}	67
**193**	$TyrO^{\bullet} + O_2^{\bullet-} \rightarrow TyrOOH$	2.49×10^{-12}	68
**194**	$TyrO^{\bullet} + AscH \rightarrow Tyr$	7.31×10^{-13}	69
**195**	$TyrO^{\bullet} + GSH \rightarrow Tyr$	3.32×10^{-15}	70
**196**	$TyrO^{\bullet} + UAH \rightarrow Tyr$	3.99×10^{-13}	68
**197**	$^{\bullet}TyrOH + O_2 \rightarrow TyrOO^{\bullet}$	1.66×10^{-18}	71
**198**	$TyrOO^{\bullet} + AscH \rightarrow TyrOOH$	1.16×10^{-14}	72
**199**	$TyrOO^{\bullet} + UAH \rightarrow TyrOOH$	3.16×10^{-15}	72
	Cell reactions		
**200**	H_2O_2 + enzymes $\rightarrow H_2O + 0.5\ O_2$ + enzymes	3.32×10^{-14}	4
**201**	$O_2^{\bullet-} + SOD \xrightarrow{2H^+} H_2O_2 + SOD$	2.65×10^{-12}	4

Table S2. Input parameters used in the KM-SUB-ELF 2.0

Parameter	Value	Unit	References
Henry's law equilibrium constant of O_3	$1.0 \cdot 10^{-2}$	M atm^{-1}	4
Henry's law equilibrium constant of H_2O_2	$9.1 \cdot 10^{4}$	M atm^{-1}	4
Henry's law equilibrium constant of $^{\bullet}OH$	29	M atm^{-1}	4
Henry's law equilibrium constant of $HO_2^{\bullet}$	$6.8 \cdot 10^{2}$	M atm^{-1}	4
Particulate mass fraction of Cu^{2+}	$3.1 \cdot 10^{-4}$	-	4
Particulate mass fraction of Fe^{2+}	$8.1 \cdot 10^{-3}$	-	4
Particulate mass fraction of quinones	$1.9 \cdot 10^{-5}$	-	4
Particulate mass fraction of SOA	0.33	-	4
Water soluble fraction of Cu^{2+}	0.40	-	4
Water soluble fraction of Fe^{2+}	0.10	-	4
Water soluble fraction of quinones	0.10	-	4
Water soluble fraction of SOA	0.10	-	4
Particulate exposure time	2	h	4
ELF catalase concentration	5	pM	4
ELF superoxide dismutase concentration	0.1	μM	See SI text
ELF glutathione concentration	108	μM	4
ELF ascorbate concentration	40	μM	4
ELF uric acid concentration	200	μM	4
ELF α-Tocopherol concentration	0.7	μM	4
ELF SP-$B_{1\text{-}25}$ concentration	$3.76 \cdot 10^{-2}$	M	8
ELF POG concentration	4.55	M	8
Lung PM accumulation time	2	h	4
PM deposition factor	0.45	-	4
Lung functional residual capacity	2750	cm^3	4
Tidal volume	1500	cm^3	4
Duration of breath	3.6	sec	4
ELF pH	7	-	4
ELF volume	20	cm^3	4
ELF surface area	$8.9 \cdot 10^{5}$	cm^2	4
ELF temperature	310	K	4
ELF O_2 concentration	$1.6 \cdot 10^{17}$	cm^3	4
Cellular concentration of H_2O_2-scavenging enzymes	1	μM	1
H_2O_2 effective membrane permeability coefficient	$1.0 \cdot 10^{-6}$	cm s^{-1}	90
H_2O_2 production in the cell layer	$1.0 \cdot 10^{14}$	cm^{-3} s^{-1}	1
$O_2^{\bullet-}$ production in the ELF layer	$2.0 \cdot 10^{14}$	cm^{-3} s^{-1}	See SI text
Thickness of cell membrane	$1.0 \cdot 10^{-4}$	cm	1
Ambient H_2O_2 concentration	1	ppb	1
Blood H_2O_2 concentration	5	μM	1
ELF alanine concentration	2531	μM	See main text
ELF arginine concentration	650	μM	See main text
ELF aspartic acid concentration	863	μM	See main text
ELF aspartate concentration	1250	μM	See main text
ELF glutamate concentration	399	μM	See main text
ELF glutamic acid concentration	1180	μM	See main text

ELF glycine concentration	2455	μM	See main text
ELF histidine concentration	219	μM	See main text
ELF isoleucine concentration	463	μM	See main text
ELF leucine concentration	92	μM	See main text
ELF lysine concentration	718	μM	See main text
ELF methionine concentration	3546	μM	See main text
ELF phenylalanine concentration	151	μM	See main text
ELF proline concentration	248	μM	See main text
ELF serine concentration	1062	μM	See main text
ELF threonine concentration	801	μM	See main text
ELF tryptophan concentration	1296	μM	See main text
ELF tyrosine concentration	49	μM	See main text
ELF valine concentration	183	μM	See main text

Table S3. Concentrations of gas-phase pollutants considered in the examined pollution scenarios (Fig. 7). The $^{\bullet}NO_2$ levels represent a 1:1 mass ratio with $PM_{2.5}$ levels[4] except for the indoor gas stove scenario, where high levels of $^{\bullet}NO_2$ have been observed.[73] Note, $PM_{2.5}$ levels are also high from cooking emissions, however, these are likely dominated by organics rather than the redox active transition metals, therefore, we use the same $PM_{2.5}$ concentration in gas stove scenario as with the indoor scenario.

	**Remote**	**Rural**	**Indoor**	**urban**	**Polluted urban**	**Indoor gas stove**
**O_3 (ppb)**	15 [74]	20 [75]	10 [76]	30 [75]	75 [77]	10 [76]
**H_2O_2 (ppb)**	0.2 [78]	0.5 [79]	0.9 [80,81]	1 [82]	2 [83]	0.9 [80,81]
**$^{\bullet}NO_2$ (ppb)**	2.1	4.2	5.3	15.9	31.9	100[73]
**$PM_{2.5}$ ($\mu g{\cdot}m^{-3}$)**	4 [84]	8 [85,86]	10 [87]	30 [75]	60 [88,89]	10 [87]

Supplementary Figures

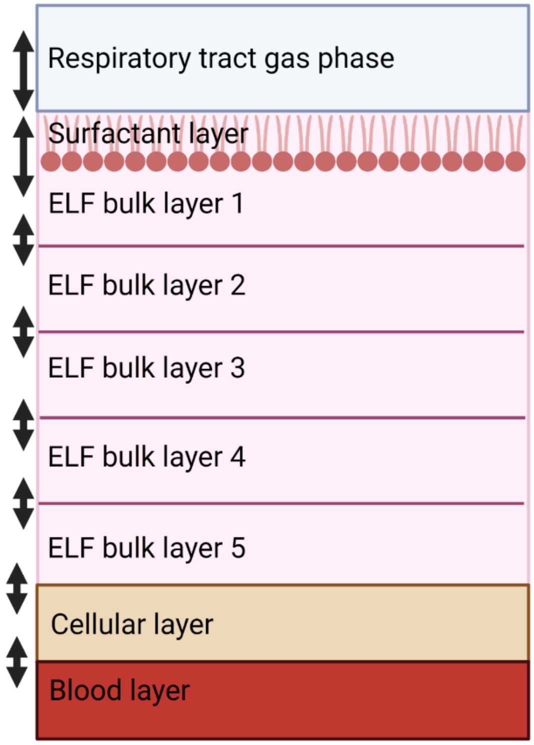

Figure S1: Schematic overview of KM-SUB-ELF 2.0. Arrows indicate mass transport between two model layers. In each layer, chemical reactions occur. Created with BioRender.com

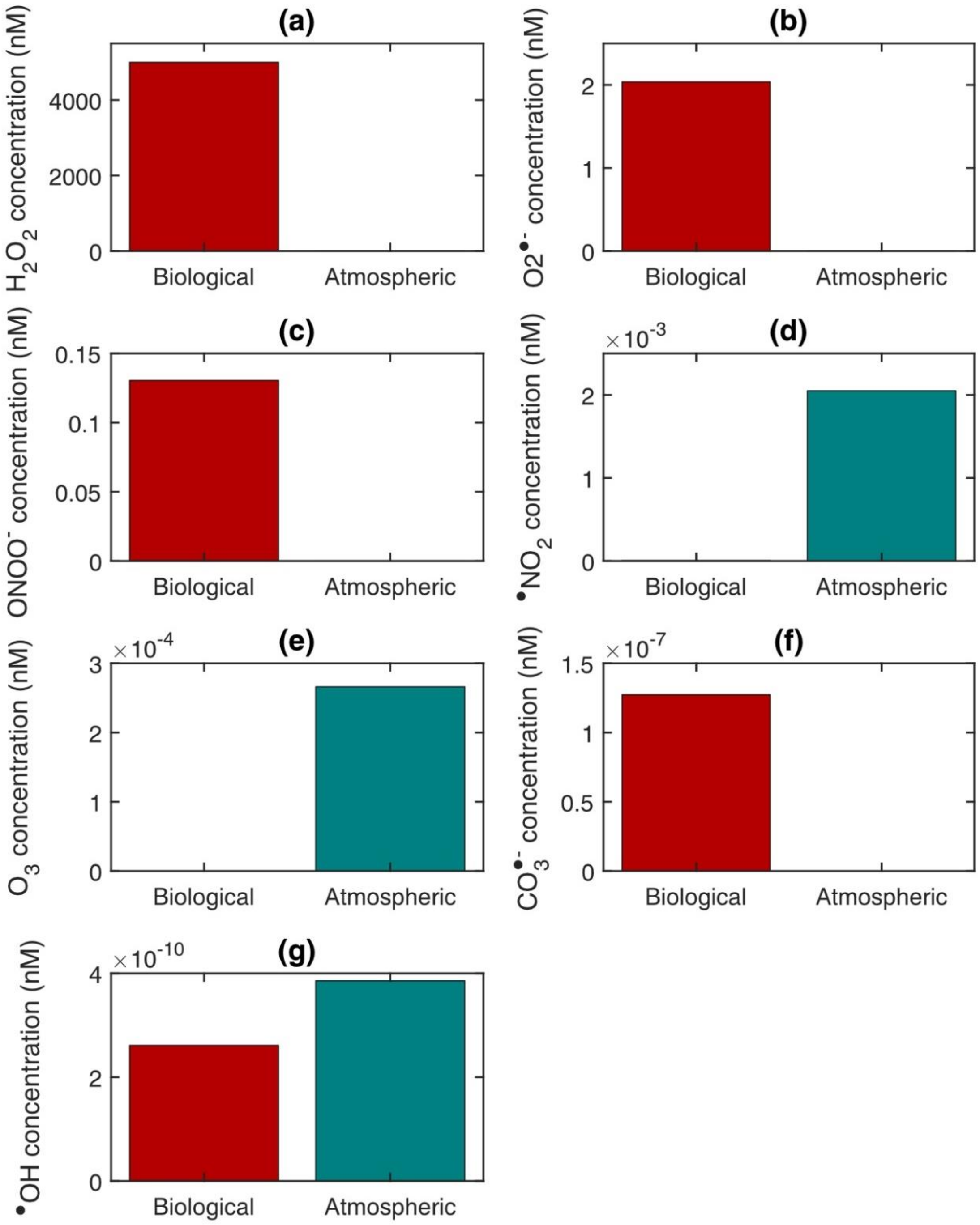

Figure S2: Biological vs. atmospheric contribution of various oxidants (a: H_2O_2, b: $O_2^{\bullet-}$, c: $ONOO^-$, d: $^{\bullet}NO_2$, e: O_3, f: $CO_3^{\bullet-}$, g: $^{\bullet}OH$) explored in the study. The biological concentrations were calculated in a model scenario where no air pollutants were present, while for the atmospheric concentrations, we use urban scenario with 30 µg m^{-3} $PM_{2.5}$ and $^{\bullet}NO_2$, as well as 30 ppb O_3.

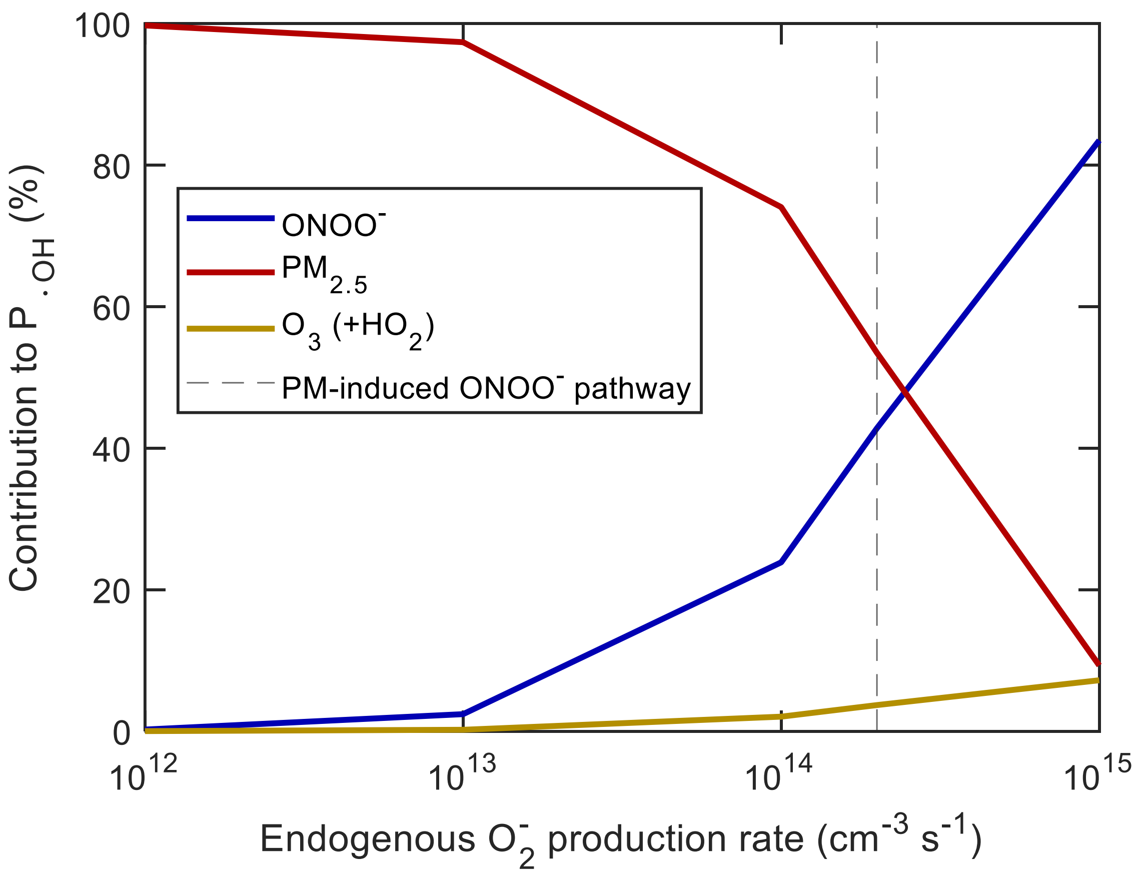

Figure S3: Contribution of different sources to $^{\bullet}OH$ production in the model as a function of endogenous superoxide production. Grey dashed line indicates the production value of unstimulated macrophages. Beyond the dashed line, the production of superoxide is emerging from PM-stimulated macrophages.

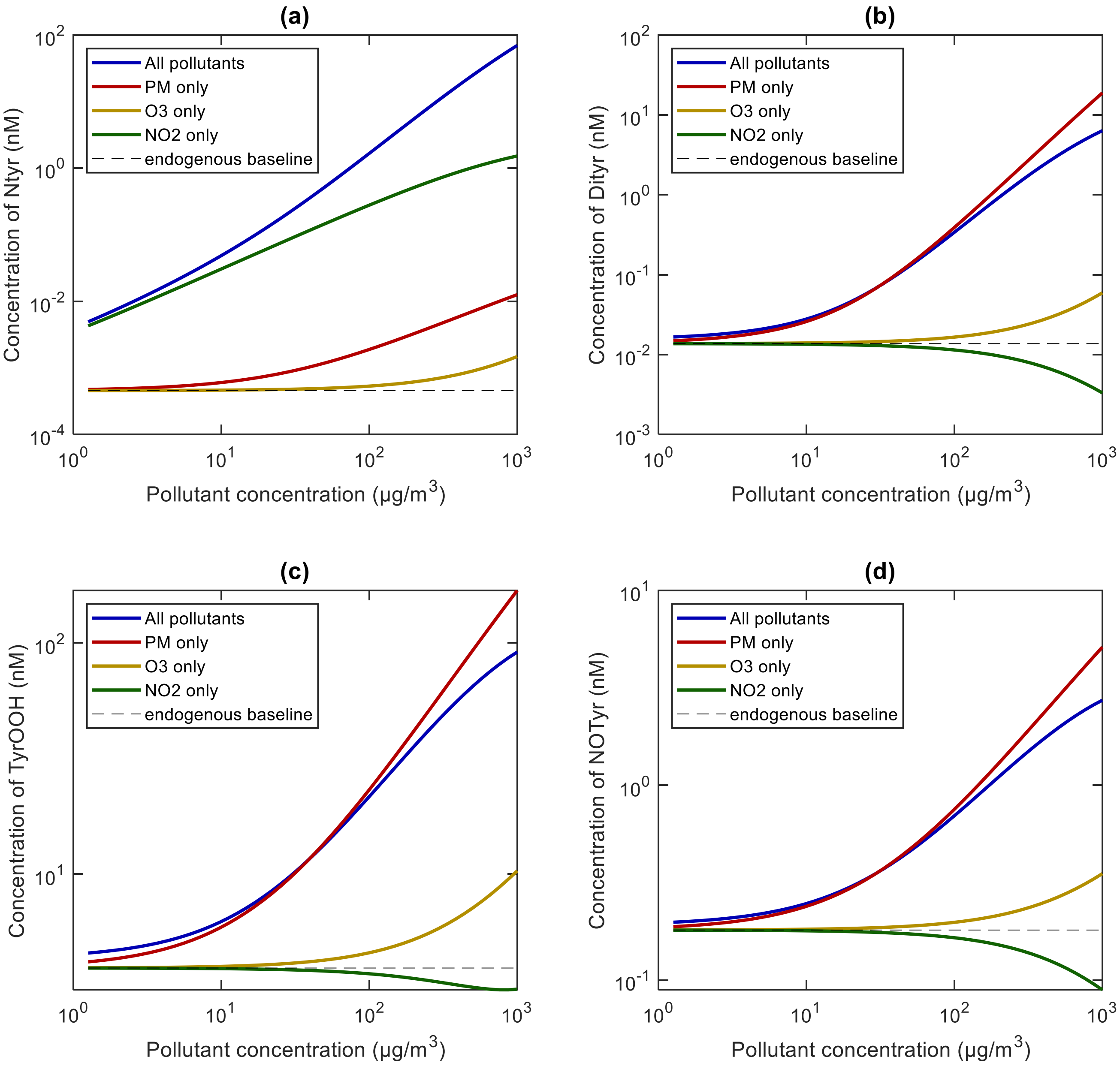

Figure S4: Concentration of modified tyrosine (a: nitrotyrosine (Ntyr), b: dityrosine (Dityr), c: tyrosine peroxide (TyrOOH), d: nitrosotyrosine (NOTyr)) in the ELF as a function of the concentration of three distinct pollutants.

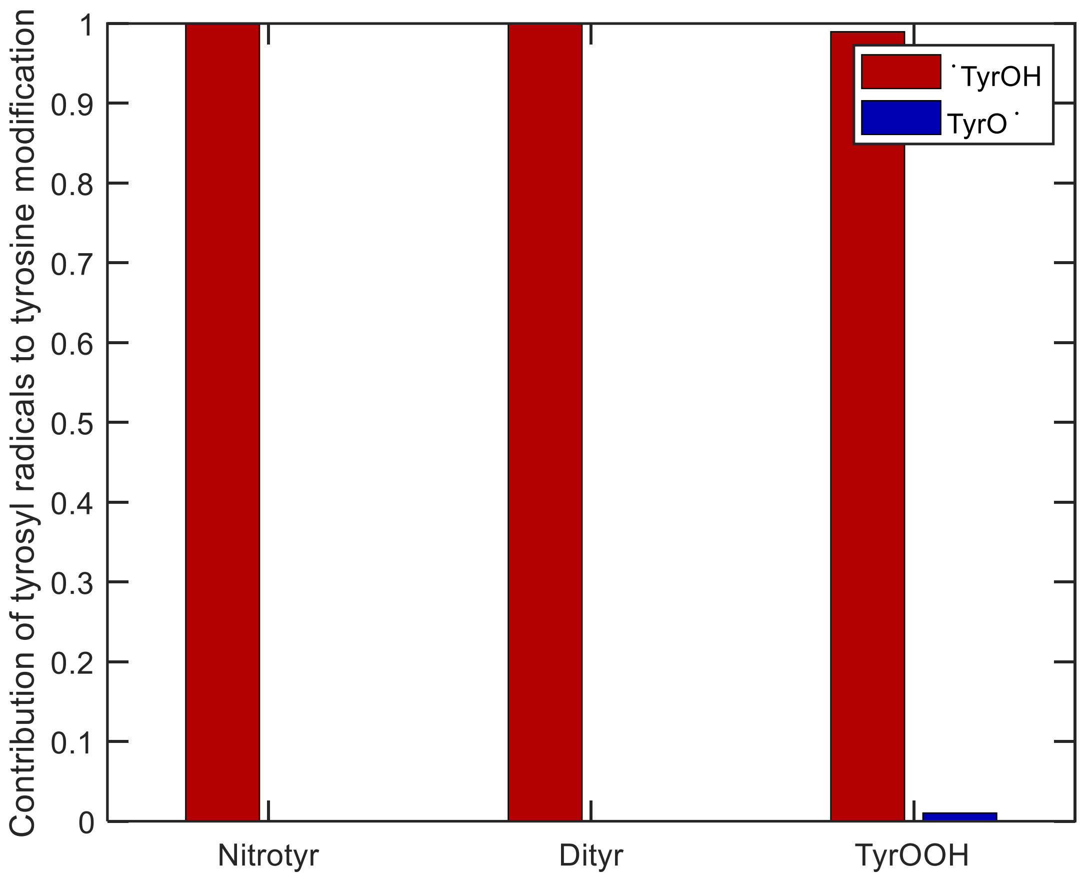

Figure S5: Fraction contribution of tyrosyl radicals to modified tyrosine considered in this study at an urban pollution scenario, of 30 µg m^{-3} $PM_{2.5}$ and $^{\bullet}NO_2$, as well as 30 ppb O_3.

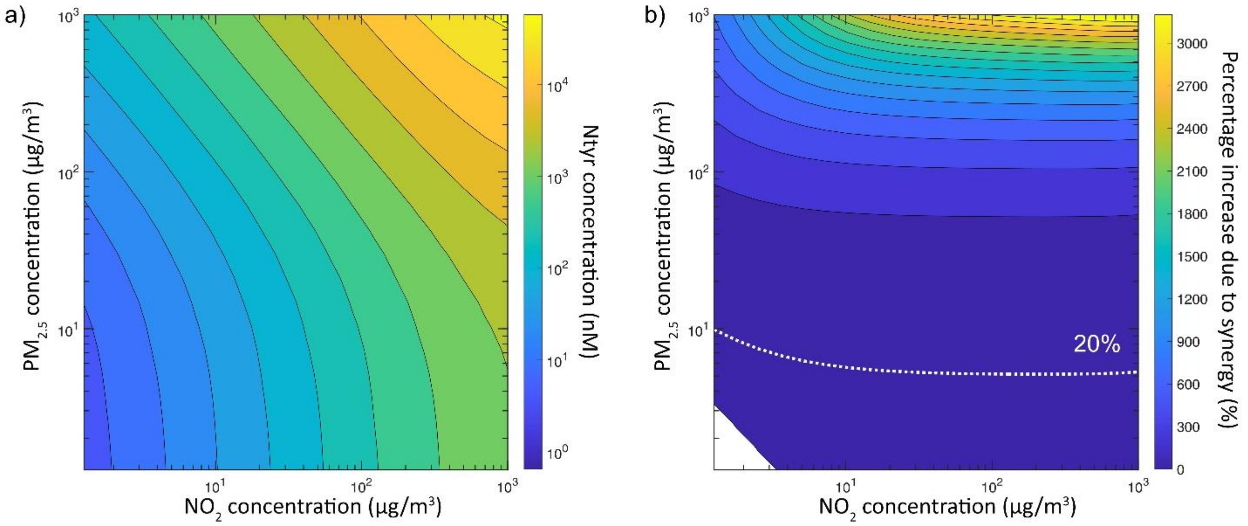

Figure S6: Nitrotyrosine concentration (a) and percentage increase in nitrotyrosine due to synergistic effects (b) in the epithelial lining fluid (ELF) after 2 h exposure as a function of $PM_{2.5}$ and $^{\bullet}NO_2$ levels. White dotted line represents the 20 % increase in synergy.

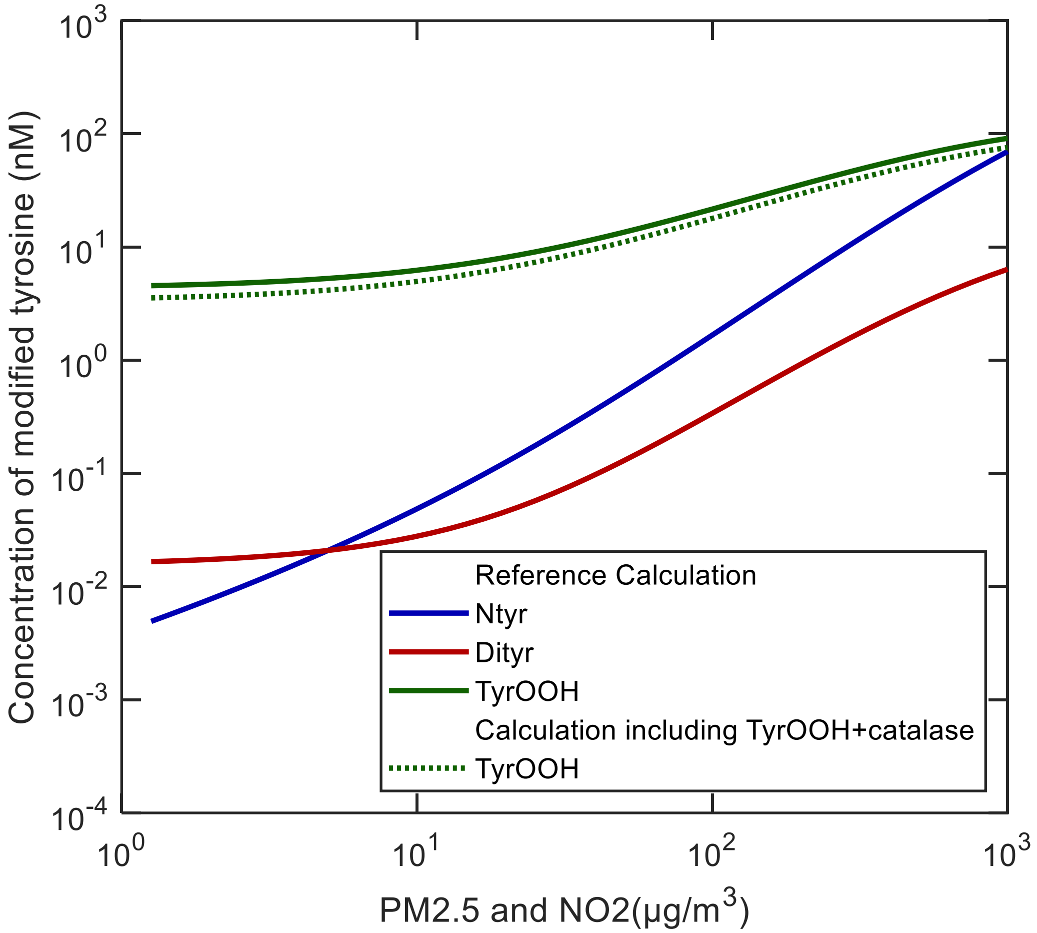

Figure S7: Concentration of modified tyrosine as a function of $PM_{2.5}$ and $^{\bullet}NO_2$. Solid lines show reference calculation results. Dotted line includes calculation where tyrosine peroxide reacts with catalase, with same reactivity as H_2O_2 to show the sensitivity of tyrosine peroxide concentration to decomposition reaction with catalase.

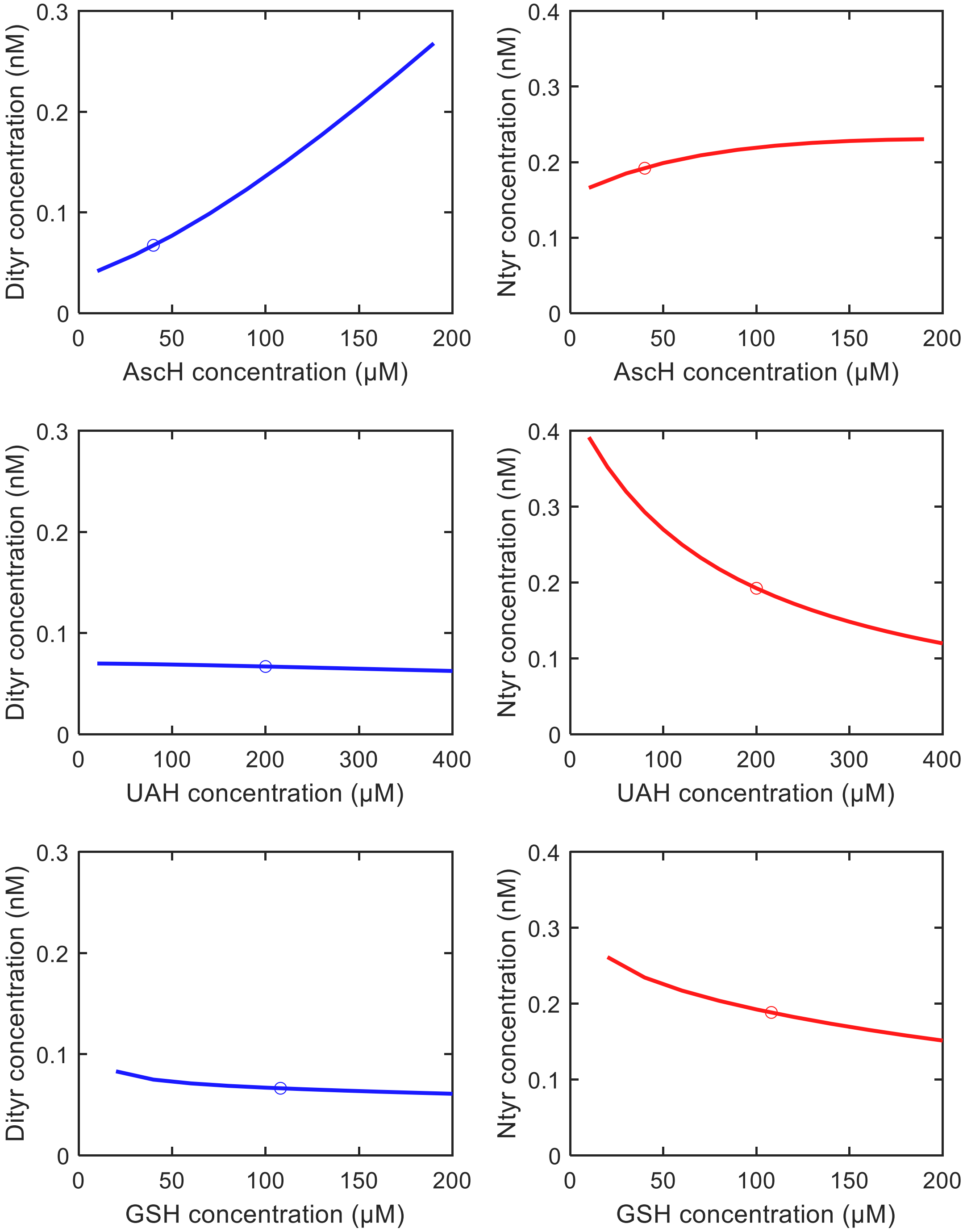

Figure S8: Concentration of Ntyr and Dityr as a function of antioxidant concentrations in a standard pollution scenario ($PM_{2.5}$ and $^{•}NO_2$: 25 μg/m^3 and 30 ppb O_3). Markers represent the values that were used in the study.

.

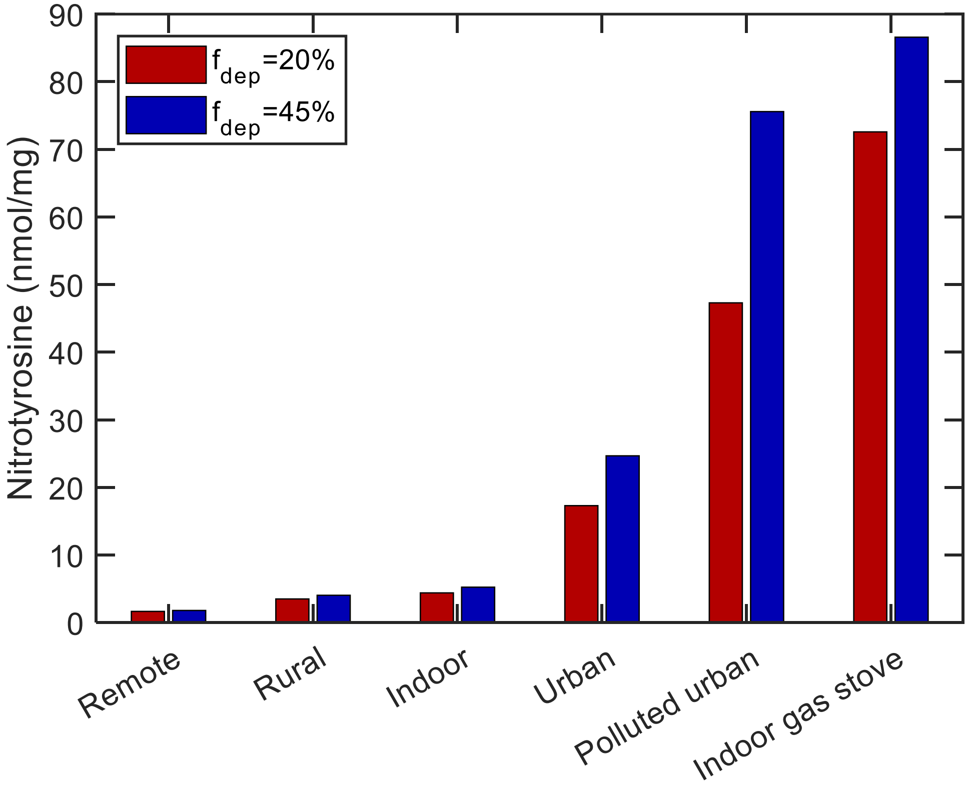

Figure S9: Sensitivity of $PM_{2.5}$ deposition fractions to concentration of Ntyr after 2 h exposure to air pollution under different pollution scenarios.

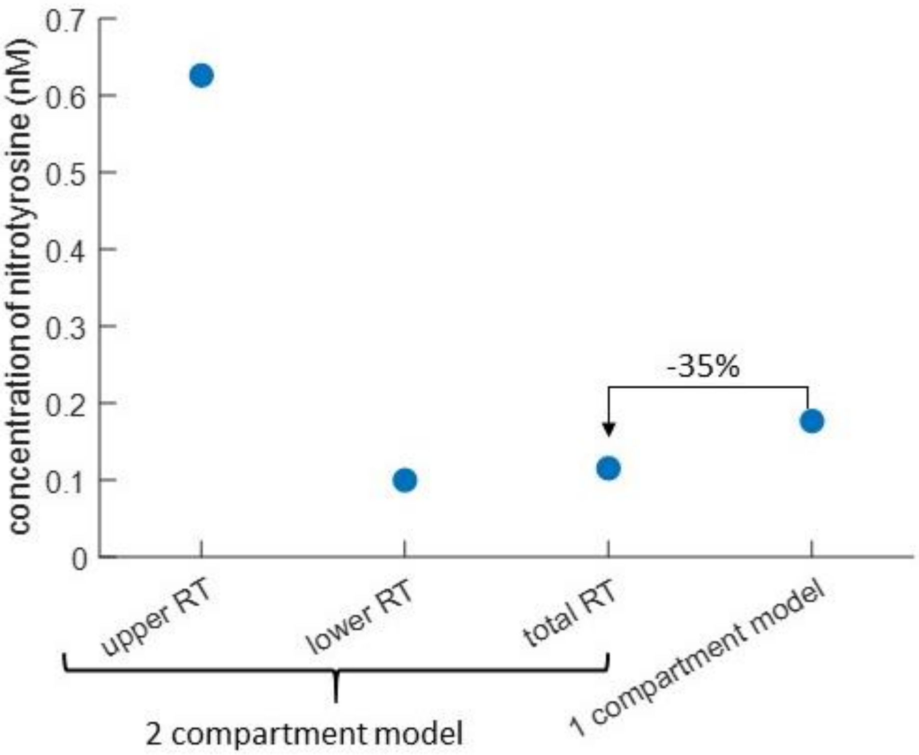

Figure S10: Concentration of Ntyr in the gas phase of the upper and lower respiratory tract (RT).

References

(1) Dovrou, E.; Lelieveld, S.; Mishra, A.; Pöschl, U.; Berkemeier, T. Influence of Ambient and Endogenous H2O2 on Reactive Oxygen Species Concentrations and OH Radical Production in the Respiratory Tract. *Environ. Sci. Atmospheres* **2023**. https://doi.org/10.1039/D2EA00179A.

(2) Saunders, S. M.; Jenkin, M. E.; Derwent, R. G.; Pilling, M. J. Protocol for the Development of the Master Chemical Mechanism, MCM v3 (Part A): Tropospheric Degradation of Non-Aromatic Volatile Organic Compounds. *Atmos. Chem. Phys.* **2003**, *3* (1), 161–180. https://doi.org/10.5194/acp-3-161-2003.

(3) Jenkin, M. E.; Saunders, S. M.; Wagner, V.; Pilling, M. J. Protocol for the Development of the Master Chemical Mechanism, MCM v3 (Part B): Tropospheric Degradation of Aromatic Volatile Organic Compounds. *Part B* **2003**, *3* (1), 181–193. https://doi.org/10.5194/acp-3-181-2003.

(4) Lelieveld, S.; Wilson, J.; Dovrou, E.; Mishra, A.; Lakey, P. S. J.; Shiraiwa, M.; Pöschl, U.; Berkemeier, T. Hydroxyl Radical Production by Air Pollutants in Epithelial Lining Fluid Governed by Interconversion and Scavenging of Reactive Oxygen Species. *Environ. Sci.* **2021**, *55* (20), 14069–14079. https://doi.org/10.1021/acs.est.1c03875.

(5) Wang, Y.; Kim, H.; Paulson, S. E. Hydrogen Peroxide Generation from α- and β-Pinene and Toluene Secondary Organic Aerosols. *Atmos. Environ.* **2011**, *45* (18), 3149–3156. https://doi.org/10.1016/j.atmosenv.2011.02.060.

(6) Tong, H.; Arangio, A. M.; Lakey, P. S. J.; Berkemeier, T.; Liu, F.; Kampf, C. J.; Brune, W. H.; Pöschl, U.; Shiraiwa, M. Hydroxyl Radicals from Secondary Organic Aerosol Decomposition in Water. *Atmos. Chem. Phys.* **2016**, *16* (3), 1761–1771. https://doi.org/10.5194/acp-16-1761-2016.

(7) Tong, H.; Lakey, P. S. J.; Arangio, A. M.; Socorro, J.; Kampf, C. J.; Berkemeier, T.; Brune, W. H.; Pöschl, U.; Shiraiwa, M. Reactive Oxygen Species Formed in Aqueous Mixtures of Secondary Organic Aerosols and Mineral Dust Influencing Cloud Chemistry and Public Health in the Anthropocene. *Faraday Discuss.* **2017**, *200*, 251–270. https://doi.org/10.1039/C7FD00023E.

(8) Lakey, P. S. J.; Berkemeier, T.; Tong, H.; Arangio, A. M.; Lucas, K.; Pöschl, U.; Shiraiwa, M. Chemical Exposure-Response Relationship between Air Pollutants and Reactive Oxygen Species in the Human Respiratory Tract. *Sci. Rep.* **2016**, *6* (1), 32916. https://doi.org/10.1038/srep32916.

(9) Cantin, A. M.; Fells, G. A.; Hubbard, R. C.; Crystal, R. G. Antioxidant Macromolecules in the Epithelial Lining Fluid of the Normal Human Lower Respiratory Tract. *J. Clin. Invest.* **1990**, *86* (3), 962–971. https://doi.org/10.1172/JCI114798.

(10) Comhair, S. A. A.; Lewis, M. J.; Bhathena, P. R.; Hammel, J. P.; Erzurum, S. C. Increased Glutathione and Glutathione Peroxidase in Lungs of Individuals with Chronic Beryllium Disease. *Am. J. Respir. Crit. Care Med.* **1999**, *159* (6), 1824–1829. https://doi.org/10.1164/ajrccm.159.6.9810044.

(11) Andreoli, S. P.; Mallett, C.; McAteer, J. A.; Williams, L. V. Antioxidant Defense Mechanisms of Endothelial Cells and Renal Tubular Epithelial Cells In Vitro: Role of the Glutathione Redox Cycle and Catalase. *Pediatr. Res.* **1992**, *32* (3), 360–365. https://doi.org/10.1203/00006450-199209000-00023.

(12) Bracaglia. HHS Public Access. *Physiol. Behav.* **2017**, *176* (3), 139–148. https://doi.org/10.1016/j.bbrc.2016.01.026.Direct.
(13) Winterbourn, C. C.; Hampton, M. B. Thiol Chemistry and Specificity in Redox Signaling. *Free Radic. Biol. Med.* **2008**, *45* (5), 549–561. https://doi.org/10.1016/j.freeradbiomed.2008.05.004.
(14) Fang, T.; Huang, Y.-K.; Wei, J.; Monterrosa Mena, J. E.; Lakey, P. S. J.; Kleinman, M. T.; Digman, M. A.; Shiraiwa, M. Superoxide Release by Macrophages through NADPH Oxidase Activation Dominating Chemistry by Isoprene Secondary Organic Aerosols and Quinones to Cause Oxidative Damage on Membranes. *Environ. Sci. Technol.* **2022**, *56* (23), 17029–17038. https://doi.org/10.1021/acs.est.2c03987.
(15) Buxton, G. V.; Greenstock, C. L.; Helman, W. P.; Ross, A. B. Critical Review of Rate Constants for Reactions of Hydrated Electrons, Hydrogen Atoms and Hydroxyl Radicals (·OH/·O^-) in Aqueous Solution. *J. Phys. Chem.* **1988**, *17* (2), 513–886. https://doi.org/10.1063/1.555805.
(16) Hoffman, M. Z.; Hayon, E. Pulse Radiolysis Study of Sulfhydryl Compounds in Aqueous Solution. *J. Phys. Chem.* **1973**, *77* (8), 990–996. https://doi.org/10.1021/j100627a005.
(17) Zhao, M. J.; Jung, L.; Tanielian, C.; Mechin, R. Kinetics of the Competitive Degradation of Deoxyribose and Other Biomolecules by Hydroxyl Radicals Produced by the Fenton Reaction. *Free Radic. Res.* **1994**, *20* (6), 345–363. https://doi.org/10.3109/10715769409145635.
(18) Kanofsky, J. R.; Sima, P. D. Reactive Absorption of Ozone by Aqueous Biomolecule Solutions: Implications for the Role of Sulfhydryl Compounds as Targets for Ozone. *Arch. Biochem. Biophys.* **1995**, *316* (1), 52–62. https://doi.org/10.1006/abbi.1995.1009.
(19) Pryor, W. A.; Giamalva, D. H.; Church, D. F. Kinetics of Ozonation. 2. Amino Acids and Model Compounds in Water and Comparisons to Rates in Nonpolar Solvents. *J. Am. Chem. Soc.* **1984**, *106* (23), 7094–7100. https://doi.org/10.1021/ja00335a038.
(20) Kim, H. I.; Kim, H.; Shin, Y. S.; Beegle, L. W.; Jang, S. S.; Neidholdt, E. L.; Goddard, W. A.; Heath, J. R.; Kanik, I.; Beauchamp, J. L. Interfacial Reactions of Ozone with Surfactant Protein B in a Model Lung Surfactant System. *J. Am. Chem. Soc.* **2010**, *132* (7), 2254–2263. https://doi.org/10.1021/ja908477w.
(21) Hasson, A. S.; Ho, A. W.; Kuwata, K. T.; Paulson, S. E. Production of Stabilized Criegee Intermediates and Peroxides in the Gas Phase Ozonolysis of Alkenes: 2. Asymmetric and Biogenic Alkenes. *J. Geophys. Res. Atmospheres* **2001**, *106* (D24), 34143–34153. https://doi.org/10.1029/2001JD000598.
(22) Hewitt, C. N.; Kok, G. L. Formation and Occurrence of Organic Hydroperoxides in the Troposphere: Laboratory and Field Observations. *J. Atmos. Chem.* **1991**, *12* (2), 181–194. https://doi.org/10.1007/BF00115779.
(23) Zhou, Z.; Abbatt, J. P. D. Formation of Gas-Phase Hydrogen Peroxide via Multiphase Ozonolysis of Unsaturated Lipids. *Environ. Sci. Technol. Lett.* **2021**, *8* (2), 114–120. https://doi.org/10.1021/acs.estlett.0c00757.
(24) Navarrete, M.; Rangel, C.; Corchado, J. C.; Espinosa-García, J. Trapping of the OH Radical by α-Tocopherol: A Theoretical Study. *J. Phys. Chem. A* **2005**, *109* (21), 4777–4784. https://doi.org/10.1021/jp050717e.
(25) Kermani, S.; Ben-Jebria, A.; Ultman, J. S. Kinetics of Ozone Reaction with Uric Acid, Ascorbic Acid, and Glutathione at Physiologically Relevant Conditions. *Arch. Biochem. Biophys.* **2006**, *451* (1), 8–16. https://doi.org/10.1016/j.abb.2006.04.015.

(26) Rush, J. D.; Bielski, B. H. J. Pulse Radiolytic Studies of the Reaction of Perhydroxyl/Superoxide O2- with Iron(II)/Iron(III) Ions. The Reactivity of HO2/O2- with Ferric Ions and Its Implication on the Occurrence of the Haber-Weiss Reaction. *J. Phys. Chem.* **1985**, *89* (23), 5062–5066. https://doi.org/10.1021/j100269a035.
(27) Christensen, H.; Sehested, K.; Corfitzen, H. Reactions of Hydroxyl Radicals with Hydrogen Peroxide at Ambient and Elevated Temperatures. *J. Phys. Chem.* **1982**, *86* (9), 1588–1590. https://doi.org/10.1021/j100206a023.
(28) Sehested, K.; Rasmussen, O. L.; Fricke, H. Rate Constants of OH with HO2,O2-, and H2O2+ from Hydrogen Peroxide Formation in Pulse-Irradiated Oxygenated Water. *J. Phys. Chem.* **1968**, *72* (2), 626–631. https://doi.org/10.1021/j100848a040.
(29) Koppenol, W. H. The Haber-Weiss Cycle – 70 Years Later. *Redox Rep.* **2001**, *6* (4), 229–234. https://doi.org/10.1179/135100001101536373.
(30) Jayson, G. G.; Parsons, B. J.; Swallow, A. J. Oxidation of Ferrous Ions by Perhydroxyl Radicals. *J. Chem. Soc. Faraday Trans. 1 Phys. Chem. Condens. Phases* **1973**, *69*, 236–242. https://doi.org/10.1039/f19736900236.
(31) Lewis, S.; Lynch, A.; Bachas, L.; Hampson, S.; Ormsbee, L.; Bhattacharyya, D. Chelate-Modified Fenton Reaction for the Degradation of Trichloroethylene in Aqueous and Two-Phase Systems. *Environ. Eng. Sci.* **2009**, *26* (4), 849–859. https://doi.org/10.1089/ees.2008.0277.
(32) Stuglik, Z.; PawełZagórski, Z. Pulse Radiolysis of Neutral Iron(II) Solutions: Oxidation of Ferrous Ions by OH Radicals. *Radiat. Phys. Chem. 1977* **1981**, *17* (4), 229–233. https://doi.org/10.1016/0146-5724(81)90336-8.
(33) Hug, S. J.; Leupin, O. Iron-Catalyzed Oxidation of Arsenic(III) by Oxygen and by Hydrogen Peroxide: PH-Dependent Formation of Oxidants in the Fenton Reaction. *Environ. Sci. Technol.* **2003**, *37* (12), 2734–2742. https://doi.org/10.1021/es026208x.
(34) Charrier, J. G.; McFall, A. S.; Richards-Henderson, N. K.; Anastasio, C. Hydrogen Peroxide Formation in a Surrogate Lung Fluid by Transition Metals and Quinones Present in Particulate Matter. *Environ. Sci. Technol.* **2014**, *48* (12), 7010–7017. https://doi.org/10.1021/es501011w.
(35) Mudway, I. S.; Kelly, F. J. Ozone and the Lung: A Sensitive Issue. *Mol. Aspects Med.* **2000**, *21* (1), 1–48. https://doi.org/10.1016/S0098-2997(00)00003-0.
(36) Masuda, T.; Shinohara, H.; Kondo, M. Reactions of Hydroxyl Radicals with Nucleic Acid Bases and the Related Compounds in Gamma-Irradiated Aqueous Solution. *J. Radiat. Res. (Tokyo)* **1978**, *16* (3), 153–161. https://doi.org/10.1269/jrr.16.153.
(37) Liphard, M.; Bothe, E.; Schulte-Frohlinde, D. The Influence of Glutathione on Single-Strand Breakage in Single-Stranded DNA Irradiated in Aqueous Solution in the Absence and Presence of Oxygen. *Int. J. Radiat. Biol.* **1990**, *58* (4), 589–602. https://doi.org/10.1080/09553009014551951.
(38) Carr, A.; Lykkesfeldt, J. *Vitamin C in Health and Disease*; MDPI-Multidisciplinary Digital Publishing Institute, 2018.
(39) Shen, J.; Griffiths, P. T.; Campbell, S. J.; Utinger, B.; Kalberer, M.; Paulson, S. E. Ascorbate Oxidation by Iron, Copper and Reactive Oxygen Species: Review, Model Development, and Derivation of Key Rate Constants. *Sci. Rep.* **2021**, *11* (1), 7417. https://doi.org/10.1038/s41598-021-86477-8.
(40) Adams, G. E.; Boag, J. W.; Currant, J.; Michael, B. D. *Absolute Rate Constants for the Reaction of the Hydroxyl Radical with Organic Compounds*; Pulse Radiolysis, 1965.

(41) Goldstein, S.; Lind, J.; Merenyi, G. Reaction of Organic Peroxyl Radicals with $^{\bullet}NO_2$ and $^{\bullet}NO$ in Aqueous Solution: Intermediacy of Organic Peroxynitrate and Peroxynitrite Species. *J. Phys. Chem. A* **2004**, *108* (10), 1719–1725. https://doi.org/10.1021/jp037431z.
(42) Jones, C. M.; Lawrence, A.; Wardman, P.; Burkitt, M. J. Electron Paramagnetic Resonance Spin Trapping Investigation into the Kinetics of Glutathione Oxidation by the Superoxide Radical: Re-Evaluation of the Rate Constant. *Free Radic. Biol. Med.* **2002**, *32* (10), 982–990. https://doi.org/10.1016/S0891-5849(02)00791-8.
(43) Winterbourn, C. C.; Metodiewa, D. The Reaction of Superoxide with Reduced Glutathione. *Arch. Biochem. Biophys.* **1994**, *314* (2), 284–290. https://doi.org/10.1006/abbi.1994.1444.
(44) Wefers, H.; Sies, H. Oxidation of Glutathione by the Superoxide Radical to the Disulfide and the Sulfonate Yielding Singlet Oxygen. *Eur. J. Biochem.* **1983**, *137* (1–2), 29–36. https://doi.org/10.1111/j.1432-1033.1983.tb07791.x.
(45) Kirsch, M.; Lehnig, M.; Korth, H.-G.; Sustmann, R.; de Groot, H. Inhibition of Peroxynitrite-Induced Nitration of Tyrosine by Glutathione in the Presence of Carbon Dioxide through Both Radical Repair and Peroxynitrate Formation. *Chem. Eur. J.* **2001**, *7* (15), 3313–3320. https://doi.org/10.1002/1521-3765(20010803)
(46) Ford, E.; Hughes, M. N.; Wardman, P. Kinetics of the Reactions of Nitrogen Dioxide with Glutathione, Cysteine, and Uric Acid at Physiological PH. *Free Radic. Biol. Med.* **2002**, *32* (12), 1314–1323. https://doi.org/10.1016/S0891-5849(02)00850-X.
(47) Wardman, P.; Sonntag, C. [3] Kinetic factors that control the fate of thiyl radicals in cells. In *Methods in Enzymology*; Elsevier: gr, 1995; Vol. 251, pp 31–45. https://doi.org/10.1016/0076-6879(95)51108-3.
(48) Luo, D.; Smith, S. W.; Anderson, B. D. Kinetics and Mechanism of the Reaction of Cysteine and Hydrogen Peroxide in Aqueous Solution. *J. Pharm. Sci.* **2005**, *94* (2), 304–316. https://doi.org/10.1002/jps.20253.
(49) Winkler, B. S.; Orselli, S. M.; Rex, T. S. The Redox Couple between Glutathione and Ascorbic Acid: A Chemical and Physiological Perspective. *Free Radic. Biol. Med.* **1994**, *17* (4), 333–349. https://doi.org/10.1016/0891-5849(94)90019-1.
(50) Buettner, G. R.; Jurkiewicz, B. A. Catalytic Metals, Ascorbate and Free Radicals: Combinations to Avoid. *Radiat. Res.* **1996**, *145* (5), 532. https://doi.org/10.2307/3579271.
(51) Alfassi, Z. B.; Huie, R. E.; Neta, P.; Shoute, L. C. T. Temperature Dependence of the Rate Constants for Reaction of Inorganic Radicals with Organic Reductants. *J. Phys. Chem.* **1990**, *94* (25), 8800–8805. https://doi.org/10.1021/j100388a011.
(52) Augusto, O.; Bonini, M. G.; Amanso, A. M.; Linares, E.; Santos, C. C. X.; De Menezes, S. L. Nitrogen Dioxide and Carbonate Radical Anion: Two Emerging Radicals in Biology. *Free Radic. Biol. Med.* **2002**, *32* (9), 841–859. https://doi.org/10.1016/S0891-5849(02)00786-4.
(53) Goldstein, S.; Czapski, G. Reactivity of Peroxynitrite versus Simultaneous Generation of $^{\bullet}NO$ and $O_2^{\bullet -}$ toward NADH. *Chem. Res. Toxicol.* **2000**, *13* (8), 736–741. https://doi.org/10.1021/tx000099n.
(54) Graetzel, M. Pulsradiolytische Untersuchung einiger Elementarprozesse der Oxydation und Reduktion des Nitritions. *Berichte Bunsenges. Fuer Phys. Chem.* **1969**, *73* (7), 646–653. https://doi.org/10.1002/bbpc.19690730707.
(55) Jacob, D. Heterogeneous Chemistry and Tropospheric Ozone. *Atmos. Environ.* **2000**, *34* (12–14), 2131–2159. https://doi.org/10.1016/S1352-2310(99)00462-8.

(56) Bonini, M. G.; Augusto, O. Carbon Dioxide Stimulates the Production of Thiyl, Sulfinyl, and Disulfide Radical Anion from Thiol Oxidation by Peroxynitrite. *J. Biol. Chem.* **2001**, *276* (13), 9749–9754. https://doi.org/10.1074/jbc.M008456200.
(57) Kurz, C. R.; Kissner, R.; Nauser, T.; Perrin, D.; Koppenol, W. H. Rapid Scavenging of Peroxynitrous Acid by Monohydroascorbate. *Free Radic. Biol. Med.* **2003**, *35* (12), 1529–1537. https://doi.org/10.1016/j.freeradbiomed.2003.08.012.
(58) Squadrito, G. L.; Cueto, R.; Splenser, A. E.; Valavanidis, A.; Zhang, H.; Uppu, R. M.; Pryor, W. A. Reaction of Uric Acid with Peroxynitrite and Implications for the Mechanism of Neuroprotection by Uric Acid. *Arch. Biochem. Biophys.* **2000**, *376* (2), 333–337. https://doi.org/10.1006/abbi.2000.1721.
(59) Briviba, K.; Kissner, R.; Koppenol, W. H.; Sies, H. Kinetic Study of the Reaction of Glutathione Peroxidase with Peroxynitrite. *Chem. Res. Toxicol.* **1998**, *11* (12), 1398–1401. https://doi.org/10.1021/tx980086y.
(60) Nauser, T.; Koppenol, W. H. The Rate Constant of the Reaction of Superoxide with Nitrogen Monoxide: Approaching the Diffusion Limit. *J. Phys. Chem. A* **2002**, *106* (16), 4084–4086. https://doi.org/10.1021/jp025518z.
(61) Lancaster, J. R. Nitroxidative, Nitrosative, and Nitrative Stress: Kinetic Predictions of Reactive Nitrogen Species Chemistry Under Biological Conditions. *Chem. Res. Toxicol.* **2006**, *19* (9), 1160–1174. https://doi.org/10.1021/tx060061w.
(62) Radi, R. Oxygen Radicals, Nitric Oxide, and Peroxynitrite: Redox Pathways in Molecular Medicine. *Proc. Natl. Acad. Sci.* **2018**, *115* (23), 5839–5848. https://doi.org/10.1073/pnas.1804932115.
(63) Sharma, V. K.; Graham, N. J. D. Oxidation of Amino Acids, Peptides and Proteins by Ozone: A Review. *Ozone Sci. Eng.* **2010**, *32* (2), 81–90. https://doi.org/10.1080/01919510903510507.
(64) Davies, M. J. The Oxidative Environment and Protein Damage. *Methionine Oxid. Methionine Sulfoxide Reductases* **2005**, *1703* (2), 93–109. https://doi.org/10.1016/j.bbapap.2004.08.007.
(65) Huie, R. E.; Shoute, L. C. T.; Neta, P. Temperature Dependence of the Rate Constants for Reactions of the Carbonate Radical with Organic and Inorganic Reductants. *Int. J. Chem. Kinet.* **1991**, *23* (6), 541–552. https://doi.org/10.1002/kin.550230606.
(66) Davies, M. J. Protein Oxidation and Peroxidation. *Biochem. J.* **2016**, *473* (Pt 7), 805–825. https://doi.org/10.1042/BJ20151227.
(67) Solar, S.; Solar, W.; Getoff, N. Reactivity of Hydroxyl with Tyrosine in Aqueous Solution Studied by Pulse Radiolysis. *J. Phys. Chem.* **1984**, *88* (10), 2091–2095. https://doi.org/10.1021/j150654a030.
(68) *Biochemistry of Peroxynitrite and Protein Tyrosine Nitration | Chemical Reviews*. https://pubs.acs.org/doi/full/10.1021/acs.chemrev.7b00568 (accessed 2023-02-03).
(69) Hunter, E. P. L.; Desrosiers, M. F.; Simic, M. G. The Effect of Oxygen, Antioxidants, and Superoxide Radical on Tyrosine Phenoxyl Radical Dimerization. *Free Radic. Biol. Med.* **1989**, *6* (6), 581–585. https://doi.org/10.1016/0891-5849(89)90064-6.
(70) Folkes, L. K.; Trujillo, M.; Bartesaghi, S.; Radi, R.; Wardman, P. Kinetics of Reduction of Tyrosine Phenoxyl Radicals by Glutathione. *Arch. Biochem. Biophys.* **2011**, *506* (2), 242–249. https://doi.org/10.1016/j.abb.2010.12.006.

(71) Candeias, L. P.; Wardman, P.; Mason, R. P. The Reaction of Oxygen with Radicals from Oxidation of Tryptophan and Indole-3-Acetic Acid. *Biophys. Chem.* **1997**, *67* (1), 229–237. https://doi.org/10.1016/S0301-4622(97)00052-5.
(72) Alfassi, Z. B.; Huie, R. E.; Kumar, M.; Neta, P. Temperature Dependence of the Rate Constants for Oxidation of Organic Compounds by Peroxyl Radicals in Aqueous Alcohol Solutions. *J. Phys. Chem.* **1992**, *96* (2), 767–770. https://doi.org/10.1021/j100181a045.
(73) Farmer, D. K.; Vance, M. E.; Abbatt, J. P. D.; Abeleira, A.; Alves, M. R.; Arata, C.; Boedicker, E.; Bourne, S.; Cardoso-Saldaña, F.; Corsi, R.; DeCarlo, P. F.; Goldstein, A. H.; Grassian, V. H.; Hildebrandt Ruiz, L.; Jimenez, J. L.; Kahan, T. F.; Katz, E. F.; Mattila, J. M.; Nazaroff, W. W.; Novoselac, A.; O'Brien, R. E.; Or, V. W.; Patel, S.; Sankhyan, S.; Stevens, P. S.; Tian, Y.; Wade, M.; Wang, C.; Zhou, S.; Zhou, Y. Overview of HOMEChem: House Observations of Microbial and Environmental Chemistry. *Environ. Sci. Process. Impacts* **2019**, *21* (8), 1280–1300. https://doi.org/10.1039/C9EM00228F.
(74) Williams, J.; Keßel, S. U.; Nölscher, A. C.; Yang, Y.; Lee, Y.; Yáñez-Serrano, A. M.; Wolff, S.; Kesselmeier, J.; Klüpfel, T.; Lelieveld, J.; Shao, M. Opposite OH Reactivity and Ozone Cycles in the Amazon Rainforest and Megacity Beijing: Subversion of Biospheric Oxidant Control by Anthropogenic Emissions. *Atmos. Environ.* **2016**, *125*, 112–118. https://doi.org/10.1016/j.atmosenv.2015.11.007.
(75) Seinfeld, J. H.; Pandis, S. N.; Noone, K. *Atmospheric Chemistry and Physics: From Air Pollution to Climate Change* ; 1998; Vol. 51. https://doi.org/10.1063/1.882420.
(76) Salonen, H.; Salthammer, T.; Morawska, L. Human Exposure to Ozone in School and Office Indoor Environments. *Environ. Int.* **2018**, *119* (May), 503–514. https://doi.org/10.1016/j.envint.2018.07.012.
(77) Yang, G.; Liu, Y.; Li, X. Spatiotemporal Distribution of Ground-Level Ozone in China at a City Level. *Sci. Rep.* **2020**, *10* (1), 1–12. https://doi.org/10.1038/s41598-020-64111-3.
(78) O'Sullivan, D. W.; Heikes, B. G.; Snow, J.; Burrow, P.; Avery, M.; Blake, D. R.; Sachse, G. W.; Talbot, R. W.; Thornton, D. C.; Bandy, A. R. Long-Term and Seasonal Variations in the Levels of Hydrogen Peroxide, Methylhydroperoxide, and Selected Compounds over the Pacific Ocean. *J. Geophys. Res. Atmospheres* **2004**, *109* (15), 1–21. https://doi.org/10.1029/2003JD003689.
(79) Aneja, V. P. Analysis of Gaseous Hydrogen Peroxide Concentrations in Raleigh, North Carolina. *Air Waste* **1994**, *44* (2), 176–183. https://doi.org/10.1080/1073161X.1994.10467247.
(80) Li, T. H.; Turpin, B. J.; Shields, H. C.; Weschler, C. J. Indoor Hydrogen Peroxide Derived from Ozone/d-Limonene Reactions. *Environ. Sci. Technol.* **2002**, *36* (15), 3295–3302. https://doi.org/10.1021/es015842s.
(81) Zhou, Z.; Abbatt, J. P. D. Formation of Gas-Phase Hydrogen Peroxide via Multiphase Ozonolysis of Unsaturated Lipids. *Environ. Sci. Technol. Lett.* **2021**. https://doi.org/10.1021/acs.estlett.0c00757.
(82) Kok, G. L.; Darnall, K. R.; Winer, A. M.; Pitts, J. N.; Gay, B. W. Ambient Air Measurements of Hydrogen Peroxide in the California South Coast Air Basin. *Environ. Sci. Technol.* **1978**, *12* (9), 1077–1080. https://doi.org/10.1021/es60145a011.
(83) He, S. Z.; Chen, Z. M.; Zhang, X.; Zhao, Y.; Huang, D. M.; Zhao, J. N.; Zhu, T.; Hu, M.; Zeng, L. M. Measurement of Atmospheric Hydrogen Peroxide and Organic Peroxides in Beijing before and during the 2008 Olympic Games: Chemical and Physical Factors

Influencing Their Concentrations. *J. Geophys. Res. Atmospheres* **2010**, *115* (17). https://doi.org/10.1029/2009JD013544.
(84) Artaxo, P.; Rizzo, L. V.; Brito, J. F.; Barbosa, H. M. J.; Arana, A.; Sena, E. T.; Cirino, G. G.; Bastos, W.; Martin, S. T.; Andreae, M. O. Atmospheric Aerosols in Amazonia and Land Use Change: From Natural Biogenic to Biomass Burning Conditions. *Faraday Discuss.* **2013**, *165*, 203–235. https://doi.org/10.1039/c3fd00052d.
(85) Clements, N.; Hannigan, M. P.; Miller, S. L.; Peel, J. L.; Milford, J. B. Comparisons of Urban and Rural PM10-2.5 and PM2.5 Mass Concentrations and Semi-Volatile Fractions in Northeastern Colorado. *Atmospheric Chem. Phys.* **2016**, *16* (11), 7469–7484. https://doi.org/10.5194/acp-16-7469-2016.
(86) Kundu, S.; Stone, Elizabeth. A. 基因的改变NIH Public Access. *Bone* **2011**, *23* (1), 1–7. https://doi.org/10.1039/c3em00719g.Composition.
(87) Patel, S.; Sankhyan, S.; Boedicker, E. K.; Decarlo, P. F.; Farmer, D. K.; Goldstein, A. H.; Katz, E. F.; Nazaroff, W. W.; Tian, Y.; Vanhanen, J.; Vance, M. E. Indoor Particulate Matter during HOMEChem: Concentrations, Size Distributions, and Exposures. *Environ. Sci. Technol.* **2020**, *54* (12), 7107–7116. https://doi.org/10.1021/acs.est.0c00740.
(88) Karambelas, A.; Holloway, T.; Kinney, P. L.; Fiore, A. M.; Defries, R.; Kiesewetter, G.; Heyes, C. Urban versus Rural Health Impacts Attributable to PM2.5 and O3 in Northern India. *Environ. Res. Lett.* **2018**, *13* (6). https://doi.org/10.1088/1748-9326/aac24d.
(89) Liu, T.; Meng, H.; Yu, M.; Xiao, Y.; Huang, B.; Lin, L.; Zhang, H.; Hu, R.; Hou, Z.; Xu, Y.; Yuan, L.; Qin, M.; Zhao, Q.; Xu, X.; Gong, W.; Hu, J.; Xiao, J.; Chen, S.; Zeng, W.; Li, X.; He, G.; Rong, Z.; Huang, C.; Du, Y.; Ma, W. Urban-Rural Disparity of the Short-Term Association of PM2.5 with Mortality and Its Attributable Burden. *The Innovation* **2021**, *2* (4), 100171. https://doi.org/10.1016/j.xinn.2021.100171.
(90) Wragg, D.; Leoni, S.; Casini, A. Aquaporin-Driven Hydrogen Peroxide Transport: A Case of Molecular Mimicry? *RSC Chem. Biol.* **2020**, *1* (5), 390–394. https://doi.org/10.1039/D0CB00160K.
